# Effect of Early Tourniquet Release on Hemolysis in Emergency Department Phlebotomy: The TOURNI-ED Randomized Trial

**DOI:** 10.3390/medicina62102009

**Published:** 2026-10-17

**Authors:** Emir Ünal, Furkan Ünal, Onatcan Ozogul, Mümin Murat Yazıcı, Eren Onur Karavin, Erhan Altunbas, Sinan Karacabey, Ali Çelik

**Affiliations:** 1 Department of Emergency Medicine, Marmara University School of Medicine, Istanbul 34899, Türkiye; emirunal@gmail.com (E.Ü.); erhnaltnbs@gmail.com (E.A.); karacabeysinan@gmail.com (S.K.); 2 Nursing Services, Marmara University Pendik Training and Research Hospital, Istanbul 34899, Türkiye; funal2800@gmail.com; 3 Department of Emergency Medicine, Marmara University Pendik Training and Research Hospital, Istanbul 34899, Türkiye; onatozogul@gmail.com (O.O.); onurkaravin@gmail.com (E.O.K.); 4 Department of Emergency Medicine, Recep Tayyip Erdoğan University Training and Research Hospital, Rize 53020, Türkiye; mmuratyazici53@gmail.com

**Keywords:** hemolysis, phlebotomy, tourniquet, emergency medicine, preanalytical quality, diagnostic reliability, blood specimen collection, randomized controlled trial

## Abstract

*Background and Objectives*: Hemolysis is the most common preanalytical error in emergency department samples and can compromise diagnostic reliability. Evidence on tourniquet-release timing under standardized pressure is limited. We assessed whether initiating cuff deflation when blood enters the first tube and completing it as that tube fills reduces hemolysis. *Materials and Methods*: In a single-center, parallel-group, 1:1 randomized trial (ClinicalTrials.gov NCT07617103), 792 adults triaged as Emergency Severity Index (ESI) 3 to 5 underwent venipuncture with stasis applied at 60 mm Hg by sphygmomanometer. Cuff deflation was initiated when blood entered the first tube and completed as that tube filled (n = 396) or after the last tube filled (n = 396). The primary outcome was the proportion of serum separator gel tubes with a hemolysis index of 1+ or greater, assessed by personnel blinded to allocation. The primary complete-case analysis included 790 participants analyzed as randomized. *Results*: Hemolysis occurred in 68 of 395 patients (17.2%) with early release and 103 of 395 (26.1%) with late release (absolute risk reduction 8.9 percentage points; 95% confidence interval (CI), 3.1–14.5; *p* = 0.003; number needed to treat (NNT) 11.3). The adjusted odds ratio in the prespecified model was 0.58 (95% CI, 0.41–0.82), and the ordinal distribution also favored early release (*p* = 0.002). No early-release patient exceeded 60 s of stasis, versus 29.0% with late release. Complication estimates were imprecise (8.1% vs. 5.6%; risk difference +2.5 points; 95% CI, −1.0 to 6.1). *Conclusions*: In adult emergency department patients presenting with non-critical acuity (ESI 3–5) undergoing direct 21-gauge venipuncture under standardized 60 mm Hg cuff pressure, early cuff release significantly reduced specimen hemolysis (NNT 11.3 for preventing one laboratory-defined hemolyzed sample). These findings cannot be generalized to resuscitated patients (ESI 1–2), intravenous catheter blood draws, winged collection devices, or uncalibrated elastic tourniquets.

## 1. Introduction

In one hospital stat laboratory, 68.2% of documented laboratory errors occurred in the preanalytical phase [1]. Preanalytical quality is a distinct domain in international quality-indicator frameworks [2].

Hemolysis is the most common preanalytical error and accounts for more than 60% of sample rejections [3]. High emergency department hemolysis rates have been reported: 25.7% among the 715 patients studied by Ersoy and Ilanbey [4], and 12.5% (95% confidence interval (CI), 11.6–13.5%) among the 4513 samples reported by Wollowitz et al. [5]. In an observational before-and-after study, Barnaby et al. reported a fall from 11.3% to 4.3% after butterfly needles were introduced [6]. A recent study from a high-volume diagnostic center documented 195 preanalytical errors over two months, recorded hemolysis and clotting as a single combined category, and found that error frequency rose on high-workload days [7].

Venous stasis during phlebotomy is a modifiable contributor to hemolysis [3]. Saleem et al. found an association between tourniquet time exceeding 1 min and higher odds of hemolysis (odds ratio 19.5; 95% CI, 5.6–67.4) [8]. Among intravenous-line draws with a recorded tourniquet time, Phelan et al. found that hemolysis rose from 10.3% to 13.9% when the tourniquet was applied for more than 60 s [9]. That threshold was exceeded in 23% of those draws. World Health Organization (WHO) guidance notes recommendations to remove the tourniquet as soon as blood flow is established [10].

The elastic tourniquet limits this literature: its applied pressure is generally not quantified in routine use, so duration and pressure cannot readily be separated. A sphygmomanometer applies stasis at a fixed, measurable pressure. Lippi et al. studied 1 and 3 min of stasis at 60 mm Hg in 23 volunteers, without the hemolysis index (HI) as an outcome [11]. In a phlebotomy training program that included releasing the tourniquet as the first tube started to fill, Lima-Oliveira et al. reduced mean tourniquet time from 118 to 30 s, outside the emergency department and without the hemolysis index as the primary outcome [12]. This trial evaluates whether release timing under standardized cuff pressure affects specimen hemolysis in emergency department patients. Within the standardized cuff-based study procedure, the randomized contrast added no equipment or materials and differed in the timing of cuff release, which also determined whether the serum separator gel tube was filled under active stasis.

The primary objective was to determine whether initiating cuff deflation at 60 mm Hg when blood enters the first tube and completing it as that tube fills, compared with maintaining it until all tubes filled, reduced the proportion of samples with a hemolysis index of 1+ or greater in green and yellow triage emergency department patients. Secondary objectives were to compare the ordinal distribution of hemolysis index categories, the proportion of samples requiring repeat venipuncture because of hemolysis, duration of venous stasis, and procedure-related complication rates.

## 2. Materials and Methods

*Design and setting.* This single-center, parallel-group, 1:1 randomized controlled superiority trial was conducted in the emergency department of a tertiary teaching hospital that evaluates approximately 70,000 adult patients annually. Patients were enrolled between 1 June and 30 July 2026. Follow-up ended with the laboratory result of the index sample. The trial was approved by the institutional clinical research ethics committee, and all participants gave written informed consent. The ClinicalTrials.gov record (NCT07617103; https://clinicaltrials.gov/study/NCT07617103 (accessed on 13 October 2026)) was first submitted on 23 May 2026, before enrollment, and first publicly posted on 1 June 2026. Reporting follows the Consolidated Standards of Reporting Trials (CONSORT) 2025 statement [13]. The final study procedures were specified before the first participant was enrolled. Refinements to the initial ethics application included the allocation method and electronic concealment, restriction of hemolysis assessment to serum separator gel specimens after laboratory consultation, specification of the secondary endpoints and multiplicity adjustment, and the operational definition of venous stasis duration. These refinements and the relationship between the initial application and final procedures are described in Section S16 of the consolidated study protocol as Supplementary Materials. No study-plan changes were made after enrollment began (Statistical Analysis Plan [SAP] Version 1.2 is an editorial update only), and there were no interim analyses and no stopping rules. Marmara University Pendik Training and Research Hospital served as the study site and was listed as the sponsor in the trial registry but had no additional role in the study design, data analysis, or decision to publish. Version 1.1 of the Statistical Analysis Plan, dated 30 May 2026, is provided alongside the current Version 1.2 as Supplementary Materials to allow direct comparison of the analysis specifications. The changes introduced in Version 1.2 are documented in its version history. A consolidated Study Protocol (Version 1.0, 8 October 2026), which also describes how the final procedures relate to the initial ethics application, is provided as Supplementary Materials.

*Participants.* Adults 18 years or older triaged as Emergency Severity Index (ESI) level 3 (yellow) or 4 to 5 (green) who required routine venipuncture were eligible. Because the intervention is defined by the timing of cuff release relative to tube filling, at least 2 collection tubes had to be ordered; patients for whom a single tube was requested were excluded. Further exclusions were critical illness or hemodynamic instability, anticoagulant or thrombolytic therapy or any condition in which collection of a coagulation tube was contraindicated, known hemolytic disease or a history of active hemolysis, red blood cell transfusion within the preceding 24 h, pregnancy of any gestational age, absence of a peripheral vein suitable for standard venipuncture, local pathology preventing collection from the antecubital fossa, blood collection from an existing intravenous catheter, inability to give informed consent, and declining to participate. Each patient could contribute only once; patients already enrolled earlier in the same visit or on a previous day were not re-enrolled, so all observations are independent. Non-critically ill adults triaged to ESI 3–5 were selected a priori to standardize baseline physiological stasis and eliminate severe hemodynamic confounding (e.g., profound shock, vasoactive drug extravasation, or catheter-induced shear stress).

Eligibility was assessed at the bedside as patients presented; a formal screening log tracking all non-enrolled presenting patients was not prospectively maintained during acute bedside enrollment due to emergency workflow constraints, so the total number of patients evaluated for eligibility and the specific breakdown of exclusions could not be quantified. No refusals to participate were recorded among the patients approached.

Venipuncture was performed by emergency department nurses, for whom bedside phlebotomy is a routine duty. All received a standardized briefing before enrollment began.

*Randomization and blinding.* The allocation sequence was generated before enrollment began by the principal investigator, who did not enroll participants or perform venipuncture, using an electronic pseudorandom number generator (Research Randomizer, randomizer.org) as a 1:1 restricted allocation schedule of 792 assignments without stratification. The sequence was implemented as 198 permuted blocks of size four, with each consecutive block containing two allocations to Group A (early release) and two to Group B (late release). To prevent selection bias and maintain unpredictability, the block size was not disclosed to clinical, nursing, or research personnel. The complete sequence was fixed before the first participant was enrolled; no sealed envelopes were used. The sequence was embedded in the electronic data collection application used at the bedside and was not accessible to the enrolling nurse; the application released a participant’s allocation only after eligibility had been confirmed and the participant had been enrolled. Descriptive checks of the implemented allocation sequence are provided in Supplementary Text S3. Participants and the nurse performing venipuncture could not be blinded: the consent process described both release timings, and the timing of cuff release is visible at the bedside. The outcome assessor (the laboratory technician recording the hemolysis index) was blinded to allocation, as was the analyst, who worked on a dataset in which the groups carried neutral codes and did not know which code corresponded to early or late release. Sequence generation, data collection, and statistical analysis were carried out by different members of the research team. Each tube carried its own laboratory information system (LIS) barcode; the serum separator gel tube was identified by that barcode alone, the allocated group appeared neither on the request nor on the tube, and the hemolysis index recorded against that barcode was the value transcribed into the data set.

*Intervention.* In both groups an aneroid sphygmomanometer of identical brand and model (ERKA; ERKA Kallmeyer Medizintechnik GmbH, Bad Tölz, Germany) was applied to the nondominant upper arm and inflated to 60 mm Hg. Venipuncture was performed with a 21-gauge (green) straight multi-sample blood collection needle and a transparent holder (Safety Blood Collection Needle, pen type; KB Medical Group Inc., Irvine, CA, USA); no winged (butterfly) sets or intravenous catheters were used. Venipuncture was performed in the antecubital fossa or, at the operator’s discretion, at an alternative peripheral site, most often the dorsum of the hand (Table 1). Tubes were drawn in the order described in the historical Clinical and Laboratory Standards Institute H3-A6 standard [14]: sodium citrate (2.7 mL), serum separator gel (5 mL), and K2-EDTA (3 mL), with any additional tubes drawn according to the treating clinician’s orders. Additional tubes were not specified by the protocol and were typically for supplementary biochemistry, a complete blood count, or troponin. Ordered on clinical grounds before allocation was revealed, their number was balanced between groups (Table 1) and was included as a covariate in the extended sensitivity model. In the early-release group cuff deflation was initiated when blood entered the first tube and was completed as that tube filled; in the late-release group cuff pressure was maintained until the last tube filled. All other aspects of the procedure were identical. No concomitant care was standardized or restricted by the protocol; all patients received routine emergency department care.

*Outcomes.* The Beckman Coulter DxC 700 AU clinical chemistry analyzer (Beckman Coulter, Inc., Brea, CA, USA) reports each serum specimen as either negative for hemolysis or as an integer hemolysis index. The primary outcome was the proportion of serum separator gel tubes with a hemolysis index of 1+ or greater, that is, free hemoglobin of 50 mg/dL or greater. The hemolysis index is produced for the serum separator gel specimen only; the citrate and K2-EDTA tubes are processed on other analyzers and carry no hemolysis index. Prespecified secondary outcomes were the ordinal distribution of the hemolysis index, the proportion of samples requiring repeat venipuncture because of hemolysis, the duration of venous stasis, and a composite complication outcome. The composite was defined as any recorded procedure-related adverse event (hematoma or ecchymosis, local swelling, oozing at the puncture site, vasovagal reaction, nerve injury, or arterial puncture) or more than 2 venipuncture attempts. Procedure-related adverse events were assessed for every patient by the nurse performing the collection, during the procedure and for up to 30 min afterwards, and recorded by type in the data collection application. Exploratory outcomes were potassium, lactate dehydrogenase, aspartate aminotransferase, and pain on a 0-to-10 visual analog scale. The exploratory analytes were measured on the same serum separator gel specimen as the hemolysis index. Prespecified subgroups were venous access difficulty, assessed before venipuncture with the 5-item modified Adult Difficult Intravenous Access (A-DIVA) scale [15] (score of 2 or greater, comprising the moderate- and high-predicted-risk categories, versus below 2), and tube count. Outcomes were not changed after the trial began. The stasis endpoint, registered as total blood collection time, is reported here as duration of venous stasis (cuff inflation to cuff release); the two coincide in the late-release group by design, as detailed in the protocol. The automated Beckman Coulter DxC 700 AU analyzer (Beckman Coulter, Inc., Brea, CA, USA) measures bichromatic absorbance and reports an internal integer hemolysis index, which the laboratory maps to categorical free hemoglobin ranges (0 to 5+); the primary endpoint was binary (HI ≥ 1+). The Mann–Whitney U comparison retained the recorded numerical HI values; the supportive ordinal regression used the reporting categories. Repeat venipuncture prompted specifically by specimen hemolysis could not be evaluated due to LIS tracking limitations, as confirmation samples ordered by clinicians after results returned were not captured.

*Sample size.* Assuming hemolysis rates of 12% with late release and 6% with early release (absolute risk reduction [ARR], 6.0 percentage points), a 2-sided alpha of 0.05, 80% power, and the normal approximation for 2 proportions with pooled variance, 356 patients per group were required. Allowing approximately 10% attrition, the target was 396 per group (792 total), and this was reached. In the trial, the observed control rate was 26.1% and the early-release rate was 17.2% (ARR 8.9 percentage points). The control rate was higher than assumed. With 395 evaluable participants per arm and this control rate, the trial had 80% power to detect an absolute risk reduction of approximately 8.2 percentage points; the observed reduction of 8.9 percentage points (95% CI, 3.1 to 14.5) was close to this threshold. Because power calculated from observed results adds no information beyond the *p* value, the confidence interval should be used to judge precision, and a smaller true effect cannot be excluded.

*Statistical analysis.* The analysis plan was specified before enrollment began. All 792 randomized participants constituted the intention-to-treat population. The primary comparison was a complete-case analysis of the 790 participants with an evaluable hemolysis index, analyzed according to randomized allocation. Risk difference, relative risk (RR), and number needed to treat (NNT) were reported with 95% CIs, using the Newcombe square-and-add Wilson method for the risk difference and the Katz logarithmic method for the risk ratio. The ordinal hemolysis index distribution, duration of venous stasis, and the exploratory continuous outcomes were compared with the Mann–Whitney U test; the proportion of patients with stasis longer than 60 s was compared with the Pearson chi-square test. The primary outcome was compared with the Pearson chi-square test with Yates continuity correction, and the composite complication outcome with the Pearson chi-square test without continuity correction. A Bonferroni-adjusted alpha of 0.0125 was applied across the 4 confirmatory secondary endpoints; exploratory analyses used alpha of 0.05 and are hypothesis-generating. The complete-case analysis is reported as primary; sensitivity analyses examined all four possible assignments of the two missing primary outcomes (Supplementary Table S2). Analyses were performed in jamovi (version 2.7.17; The jamovi project, Sydney, Australia), based on R version 4.5. Several analyses go beyond the plan and are reported as supportive rather than confirmatory: the within-group association between stasis duration and hemolysis, alternative collapsings of the ordinal scale, the proportional odds ordinal regression, the relation between stasis duration and tube count, and the 45 s stasis threshold (the plan specified only 60 s). The secondary-endpoint effect estimates are likewise supportive, because the plan specified interval methods for the primary outcome only. The primary statistical inference was prespecified as the unadjusted complete-case comparison using the continuity-corrected Pearson chi-square test. In accordance with the study protocol and SAP, a supportive multivariable logistic-regression model was prespecified to adjust for determinants of venous access difficulty (modified A-DIVA score, triage category, and number of venipuncture attempts); adjustment was intended to improve precision and did not replace the unadjusted primary comparison. We additionally fitted an extended model that included routinely collected baseline and procedural variables (age, sex, body mass index, nurse experience, and number of tubes collected) together with A-DIVA score and triage category. These additional covariates were not listed in the SAP, so this model is reported as a sensitivity analysis. Continuous variables were entered as linear terms. Operator identity was not recorded; nurse experience (years) was the only operator-level characteristic available. Both models yielded consistent effect estimates. A cumulative logit proportional odds ordinal logistic-regression model was additionally fitted across the six laboratory hemolysis categories (negative, 1+, 2+, 3+, 4+, and 5+ or greater) to estimate the odds ratio for shifting into higher hemolysis categories. The Mann–Whitney U test was retained as the primary ordinal comparison because it is distribution-free and does not require the proportional odds assumption; the ordinal regression was not prespecified and is reported as supportive.

*Use of generative artificial intelligence.* Claude (Anthropic; Sonnet 5.0) assisted in the development of the electronic data collection application and language editing. OpenAI image generation was used to create the graphical abstract. Statistical analysis was performed by E.A. and A.Ç. on the neutrally coded dataset, and the principal investigator reviewed the results only after database lock; analyses used jamovi.

*Patient and public involvement.* Patients and the public were not formally involved in the design, conduct, or reporting of this trial. Findings will be disseminated to the emergency department and laboratory teams of the participating center and through peer-reviewed publication.

## 3. Results

Of 792 patients randomized, 396 were allocated to each group; all received the allocated intervention and none were lost to follow-up (Figure 1). All randomized participants constituted the intention-to-treat population. No deviation from the allocated release timing occurred, so restricting the primary complete-case analysis to participants who adhered to the intervention did not change its result. The primary outcome was evaluable in 790 patients, 395 per group, who were analyzed according to randomized allocation. The hemolysis index was unavailable for one patient per group because the laboratory invalidated the specimen; both were among the 12 patients with a specimen rejected by the laboratory during the study. Groups were balanced at baseline (Table 1). Specimen rejection and evaluability: Twelve patients had at least one laboratory-rejected tube (five in the early-release group and seven in the late-release group). For 10 patients, rejection involved a non-primary auxiliary tube (citrate or EDTA clotted or underfilled), whereas the primary serum separator gel tube was intact and evaluable for hemolysis. Only two patients had an invalid primary serum tube (Patient 394 in late release due to a barcode mismatch; Patient 569 in early release due to insufficient serum volume), accounting for the two missing primary outcomes (395 evaluable per group). All serum biochemistry analyses used the 790 participants of the primary analysis population (Supplementary Table S4).

**Primary outcome.** Hemolysis occurred in 68 of 395 patients (17.2%) with early release and 103 of 395 (26.1%) with late release, an absolute reduction of 8.9 percentage points (95% CI, 3.1–14.5; *p* = 0.003; Table 2). In the prespecified model adjusted for total A-DIVA score, triage category, and number of venipuncture attempts, the adjusted odds ratio was 0.580 (95% CI, 0.409–0.822; *p* = 0.002; Table 2). The extended sensitivity model yielded a consistent estimate (0.587; 95% CI, 0.413–0.834; Supplementary Table S1). Across all four possible assignments of the two missing outcomes, the absolute risk reduction ranged from 8.59 to 9.09 percentage points, and the conclusion was unchanged (Supplementary Table S2). In a tipping-point sensitivity analysis imputing missing early-release outcomes as hemolyzed and missing late-release outcomes as negative, the absolute risk reduction remained statistically significant (ARR 8.59%; 95% CI, 2.84–14.27; *p* = 0.004).

**Duration of venous stasis and protocol adherence.** Stasis was 20 s shorter with early release (*p* < 0.001; Table 3). This difference follows from the definition of the intervention and is reported as an indicator of protocol fidelity rather than as a benefit of early release. No patient in the early-release group exceeded 60 s of stasis and only 25 (6.3%) exceeded 45 s, whereas in the late-release group 115 patients (29.0%) exceeded 60 s (0/396 vs. 115/396; Pearson chi-square, *p* < 0.001) and 362 (91.4%) exceeded 45 s. Exploratory analyses of stasis duration, tube count, and hemolysis are provided in Supplementary Text S3.

**Secondary and exploratory outcomes.** The ordinal distribution of the hemolysis index favored early release (*p* = 0.002; Table 3). Exploratory checks using alternative ordinal categorizations are reported in Supplementary Text S3. The composite complication outcome was numerically more frequent with early release, with an imprecise between-group estimate (Table 3). The prespecified proportion of hemolyzed samples requiring repeat venipuncture proved unmeasurable. The data collection form captured only repeat collections prompted by laboratory rejection of a tube other than the serum separator gel tube: 10 such repeat collections were recorded, all for clotting, underfilling, or insufficient volume. Over the study period the laboratory rejected specimens from 12 patients in total (five in early release and seven in late release), none for hemolysis. The two rejections affecting the primary serum separator gel tube were a barcode mismatch in the late-release group (Patient 394) and insufficient serum volume in the early-release group (Patient 569), accounting for the two missing primary-outcome measurements (Supplementary Table S4). For these two patients, biochemistry from a second serum separator tube of the same draw was available but was excluded from all biochemical analyses, which therefore used the same 790 patients. Confirmation samples ordered by clinicians after results returned were not recorded, so no between-group comparison could be performed. In the proportional odds ordinal logistic-regression model, early release was associated with a 41.6% reduction in the odds of higher hemolysis categories (odds ratio 0.584; 95% CI, 0.415–0.822; *p* = 0.0021).

**Table 3:** Prespecified secondary endpoints, harms by category, and exploratory analyses.

Outcome	Early-Release Group (n = 396)	Late-Release Group (n = 396)	Effect Size (95% CI)	*p* Value
**Confirmatory secondary endpoints (Bonferroni-adjusted α = 0.0125)**				
Secondary endpoint 1. Ordinal hemolysis index distribution †	—	—	—	0.002
Supportive binary contrast: HI ≥ 2+, n/N (%)	20/395 (5.1)	38/395 (9.6)	RD −4.6 (−8.3 to −0.9); RR 0.53 (0.31 to 0.89)	—
Negative, n (%)	327 (82.8)	292 (73.9)		
1+, n (%)	48 (12.2)	65 (16.5)		
2+, n (%)	11 (2.8)	24 (6.1)		
3+, n (%)	2 (0.5)	5 (1.3)		
4+ or greater, n (%)	7 (1.8)	9 (2.3)		
Secondary endpoint 2. Hemolyzed sample → repeat blood draw	Unmeasurable	Unmeasurable	—	—
Secondary endpoint 3. Duration of venous stasis, s—median (IQR)	35 (32–39)	57 (51–61)	−20 s (−21 to −19) §	<0.001
Secondary endpoint 4. Composite complication outcome, n (%)	32 (8.1)	22 (5.6)	2.5 (−1.0 to 6.1); RR 1.45 (0.86 to 2.46)	0.159
Patients with ≥1 recorded adverse event, n (%)	16 (4.0)	10 (2.5)	1.5 (−1.1 to 4.2); RR 1.60 (0.74 to 3.48)	0.232
**Recorded procedure-related adverse events, by type (descriptive)**				
Hematoma	6 (1.5)	7 (1.8)		
Ecchymosis	5 (1.3)	1 (0.3)		
Local swelling at the puncture site	3 (0.8)	0 (0)		
Oozing at the puncture site	0 (0)	2 (0.5)		
Vasovagal reaction	2 (0.5)	1 (0.3)		
Nerve injury or paresthesia	0 (0)	0 (0)		
Arterial puncture	0 (0)	0 (0)		
Cellulitis or phlebitis	0 (0)	0 (0)		
Seizure	0 (0)	0 (0)		
Total events recorded, n	16	11		
**Procedural difficulty (contributes to the composite; not classified as a harm)**				
>2 venipuncture attempts, n (%)	18 (4.5)	15 (3.8)	0.8 (−2.1 to 3.7); RR 1.20 (0.61 to 2.35)	0.594
**Exploratory analyses (no correction, α = 0.05)**				
Potassium, mmol/L—median (IQR)	4.3 (4.0–4.5)	4.2 (4.0–4.5)	—	0.470
LDH, U/L—median (IQR)	170.0 (149.5–206.1)	168.0 (147.8–209.5)	—	0.494
AST, U/L—median (IQR)	21.0 (17.4–29.7)	21.2 (17.6–27.9)	—	0.995
VAS pain score—median (IQR)	2.0 (1.0–3.0)	2.0 (1.0–3.0)	—	0.937

Note. A Bonferroni-adjusted α of 0.0125 was applied across the 4 confirmatory secondary endpoints. The composite complication outcome was defined as any recorded procedure-related adverse event (hematoma or ecchymosis, local swelling, oozing at the puncture site, vasovagal reaction, nerve injury, or arterial puncture) or more than 2 venipuncture attempts; its components are indented beneath it and are not mutually exclusive. Twenty-six patients had at least 1 recorded event and 27 events were recorded in total, because 1 late-release patient had both a vasovagal reaction and a hematoma. Individual adverse event types are reported descriptively, without between-group significance testing, because the trial was not powered to detect differences in complication rates. *p* values for the confirmatory endpoints are from the Mann–Whitney U test (secondary endpoints 1 and 3) and the Pearson chi-square test without continuity correction (secondary endpoint 4); the same test was used for the number of patients with at least 1 recorded adverse event and for more than 2 venipuncture attempts. Exploratory analyses were compared with the Mann–Whitney U test. Duration of venous stasis was measured from cuff inflation to cuff release; this endpoint was registered as total blood collection time, and in the late-release group the two are identical by design. More than 2 venipuncture attempts contributes to the prespecified composite but is reported separately as a measure of procedural difficulty rather than as a harm. No correction was applied to exploratory analyses, which should be interpreted as hypothesis-generating. † Denominators are 396 per group, except for the ordinal hemolysis index (secondary endpoint 1; 395 evaluable specimens per group, with percentages of 395) and for potassium, LDH, AST, and VAS pain score, which were analyzed in the 790 patients of the primary analysis population (395 per group; Supplementary Table S4). The laboratory reports 6 categories by free hemoglobin concentration (negative, <50 mg/dL; 1+, 50–99; 2+, 100–199; 3+, 200–299; 4+, 300–500; 5+, >500 mg/dL). For categorical presentation and the supportive ordinal model, recorded HI values above 5 were grouped in the highest category (5+). The top 2 are combined here as 4+ or greater because of small numbers. The Mann–Whitney U test used the recorded numerical HI without top-coding; categorical presentation and the supportive ordinal model used the reporting categories. AST = aspartate aminotransferase; LDH = lactate dehydrogenase; VAS = visual analog scale. Effect sizes compare the early-release group with the late-release group; the risk difference (RD) is in percentage points, from the Newcombe square-and-add Wilson method, and the risk ratio (RR) from the Katz logarithmic method. The ordinal endpoint was tested with the Mann–Whitney U test and has no single effect measure. The separately displayed HI ≥ 2+ contrast is supportive rather than a prespecified confirmatory endpoint; no *p* value is reported for that contrast. § Hodges-Lehmann estimate of the between-group location shift (early minus late), with the Moses confidence interval.

Potassium, lactate dehydrogenase, aspartate aminotransferase, and pain scores did not differ between groups (Table 3). Analyzed by hemolysis status rather than by group, lactate dehydrogenase exceeded 250 U/L in 67 of 171 hemolyzed samples (39.2%) versus 17 of 619 nonhemolyzed samples (2.7%). Aspartate aminotransferase exceeded 40 U/L in 45 of 171 (26.3%) versus 39 of 619 (6.3%).

**Subgroup analyses.** No statistically significant interaction was detected between treatment allocation and venous access difficulty (interaction *p* = 0.54) or tube count (interaction *p* = 0.31), although the trial had limited statistical power to rule out modest effect heterogeneity across subgroups (Figure 2). Hemolysis was more frequent with an A-DIVA score of 2 or greater in both arms.

## 4. Discussion

Initiating cuff deflation at 60 mm Hg when blood entered the first tube and completing it as that tube filled reduced hemolysis from 26.1% to 17.2% in green and yellow triage emergency department patients, preventing roughly one hemolyzed sample for every 11 patients. Covariate adjustment yielded a similar association, and the ordinal hemolysis index distribution also favored early release. Subgroup analyses did not detect evidence of heterogeneity but did not establish a uniform effect across subgroups. The number needed to treat was 11.3 (95% CI, 6.9–32.1), representing the number of patients managed with early release required to prevent one additional laboratory-defined hemolyzed blood sample (HI ≥ 1+) under standardized study conditions.

Measured stasis duration supported intervention fidelity. Previous observational studies associated longer tourniquet application with more frequent hemolysis [8,9]. The present findings support evaluating release timing as a modifiable procedural factor, but do not establish a universal duration threshold or show that early release will prevent prolonged stasis in every clinical setting.

Prior stasis research documented effects of stasis on measured biochemical and hematologic analytes in volunteers [11,16] and the feasibility of shortening tourniquet time within a training protocol [12], but neither addressed emergency department hemolysis directly. Release timing altered two interconnected procedural elements simultaneously: the total duration of venous stasis and whether the serum separator gel tube was itself drawn under active stasis. Because that tube was second in the collection sequence, it filled without active stasis in the early-release group (0 mm Hg) but under ongoing 60 mm Hg stasis in the late-release group. The trial evaluates this composite procedural strategy as a practical bundle, and the individual contributions of stasis duration versus draw-under-stasis cannot be isolated. The procedural contrast delivered by the intervention naturally varied with ordering practice: the difference in stasis duration was approximately 12 s in the 3-tube stratum and 24 s in the 4-tube stratum. While the interaction test did not demonstrate statistically significant heterogeneity (*p* = 0.31), the point estimate for risk reduction was numerically greater among patients requiring four collection tubes (RR 0.58 vs. 0.87). Given the limited statistical power of the interaction test, this exploratory observation is hypothesis-generating only and is equally consistent with chance.

Collection technique is an important determinant of specimen hemolysis. In the systematic review by Heyer et al., straight-needle venipuncture was associated with less hemolysis than intravenous catheter collection (pooled risk ratio, 0.16; 95% CI, 0.11–0.24); antecubital placement also favored lower hemolysis rates in catheter draws [17]. Emergency department studies likewise show associations between collection-device selection and hemolysis [4,5]. All participants in the present trial underwent direct venipuncture with a straight steel multi-sample needle. The observed difference therefore concerns release timing within that collection technique, rather than a comparison of collection devices. Structured phlebotomy training can improve procedural conformity [18], and all participating nurses received a standardized briefing.

**Nature of the outcome.** The primary outcome is a preanalytical quality indicator, not a patient-centered event. Hemolysis was common (171 of 790 valid samples), yet none of the laboratory rejections described in the Results was for hemolysis. This reflects institutional policy: the laboratory reports hemolyzed samples with an alert rather than rejecting them, leaving the decision to redraw with the clinician. Substantial inter-institutional differences in how hemolysis is detected, reported, and acted on are well documented [19,20]. The observed reduction in hemolysis therefore cannot be extrapolated to reductions in diagnostic delay, repeat phlebotomy, hospital costs, or clinical misdiagnosis, none of which were measured in this trial.

The potential diagnostic relevance of hemolysis extends beyond specimen rejection. Median lactate dehydrogenase was 163 U/L in the 619 specimens reported as negative and 345 U/L in the 23 specimens with an index of 3+ or greater; corresponding aspartate aminotransferase values were 20.7 and 42.0 U/L. These descriptive comparisons by hemolysis status are distinct from the randomized treatment comparison and do not establish a clinical benefit of early release. In one patient without known renal failure, a potassium concentration of 7.1 mmol/L in a heavily hemolyzed specimen was interpreted as pseudohyperkalemia; the electrocardiogram showed sinus rhythm without T-wave changes, and the repeat sample yielded 4.8 mmol/L. This observation illustrates a possible consequence of specimen interference, but the trial did not systematically measure hemolysis-related clinical decisions or subsequent testing.

Safety. The composite complication outcome occurred in 8.1% of early-release and 5.6% of late-release participants (risk difference, +2.5 percentage points; 95% CI, −1.0 to 6.1; Table 3). Because the 95% confidence interval includes an excess risk of up to 6.1 percentage points and the trial was not powered for safety equivalence, the non-significant *p* value must not be interpreted as proof of safety. Early release provides only limited short-term safety evidence. Recorded events included hematoma, ecchymosis, local swelling, puncture-site oozing, and vasovagal reactions. When the composite was restricted to harm components alone (excluding more than two venipuncture attempts), the between-group difference remained imprecise (4.0% vs. 2.5%); together with unblinded event ascertainment, this further limits interpretation of the safety comparison.

### 4.1. Limitations

The trial measured a preanalytical quality indicator and was not designed to capture diagnostic delay, length of stay, or cost, so whether the observed difference translates into patient-centered outcomes is unknown. The sample size assumed a control-group hemolysis rate of 12%, whereas the observed rate was 26.1%; the prespecified sample size was not revised at any point. The high observed control rate likely reflects the sensitive threshold defining the primary outcome: a hemolysis index of 1+ corresponds to free hemoglobin of 50 mg/dL or greater, a definition that also captures low-grade hemolysis. The number needed to treat therefore applies to laboratory-defined hemolysis at this threshold and cannot be transferred directly to settings using different hemolysis thresholds or reporting policies. The prespecified secondary outcome of repeat venipuncture due to hemolysis was unmeasurable rather than zero, because confirmation samples ordered after results returned were not recorded; future studies should match hemolysis-alert results in the laboratory information system to subsequent repeat orders for the same patient.

The trial was conducted at a single center, and hemolysis rates and sample-acceptance policies vary between institutions. Stasis was applied with a sphygmomanometer at a fixed 60 mm Hg, which isolated timing from pressure. Elastic tourniquets generate an unmeasured and variable pressure, so the size of the effect reported here should not be assumed to transfer to them unchanged. Straight needles were not compared with winged sets. Because early release altered both the total duration of stasis and whether the serum tube was filled under stasis, the effect cannot be attributed to release timing alone. Because no screening log was prospectively maintained during acute bedside enrollment, the number of patients assessed for eligibility and the specific reasons for exclusion could not be quantified. Although no refusals were recorded among the patients approached, the participation rate cannot be reported, and selection bias at the point of enrollment remains a theoretical limitation.

Blinding of the nurse performing venipuncture was impossible; measurement was automated on the Beckman Coulter DxC 700 AU clinical chemistry analyzer (Beckman Coulter, Inc., Brea, CA, USA). Because the nurse performing venipuncture could not be blinded, operator-controlled factors such as aspiration force and needle manipulation leave a potential performance bias channel that blinding of laboratory measurement does not address. Balanced procedural proxies between groups (first-attempt success 87.4% vs. 87.9%; more than two attempts 4.5% vs. 3.8%) argue against systematic differences in technique, although unrecorded micro-behaviors cannot be excluded. Allocation followed the 198 permuted blocks of size four described in Materials and Methods; the block size was not disclosed to clinical, nursing, or research personnel. Each patient’s allocation was corroborated by the recorded duration of stasis. Operator identity was not recorded, so clustering by phlebotomist could not be assessed; nurse experience was included in the extended sensitivity model but is not a substitute. Enrollment was concentrated in daytime and evening shifts, so patients presenting overnight are not represented. Randomization was concurrent within each shift. Adverse events were monitored only during phlebotomy and the subsequent 30 min observation window. Delayed complications, such as slowly evolving ecchymosis or late-onset hematoma occurring after discharge, were not captured; thus, the reported rates reflect early procedural complications rather than total cumulative incidence. Procedural adverse events were recorded by the unblinded nurse performing venipuncture. For soft endpoints such as hematoma or ecchymosis, the absence of assessor blinding leaves the complication estimates susceptible to ascertainment bias, the direction of which cannot be predicted. The complication findings should therefore be interpreted as exploratory.

The intervention standardized the cue for cuff release, not a fixed duration of stasis. In the comparator group, stasis duration increased with the number of tubes ordered. The contrast the trial delivered was therefore 12 s in the 3-tube stratum and 24 s in the 4-tube stratum, so the benefit should be expected to vary with local ordering practice, and a dose–response relationship could not be separated from allocation. The effect may also depend on the position of the primary-outcome tube in the collection order. Ascertainment was identical in both groups.

### 4.2. Clinical Implications

Under the standardized cuff-based conditions studied, initiating cuff deflation when blood entered the first tube and completing it as that tube filled reduced specimen hemolysis without requiring additional equipment or consumables. First-tube filling provides a practical procedural cue within the existing collection workflow. This finding is relevant to emergency department phlebotomy quality improvement, but implementation in other settings should account for collection devices, tube order, patient selection, and local laboratory reporting practices.

## 5. Conclusions

In this single-center randomized trial of adult emergency department patients presenting with non-critical acuity (ESI 3–5) undergoing direct 21-gauge venipuncture under standardized 60 mm Hg cuff pressure, initiating cuff deflation when blood entered the first tube and completing it as that tube filled significantly reduced specimen hemolysis (NNT 11.3 for laboratory-defined hemolysis). These findings apply to standardized direct venipuncture and cannot be extrapolated to critically ill patients (ESI 1–2), intravenous catheter collections, winged devices, or uncalibrated elastic tourniquets. Multicenter studies should determine applicability across diverse clinical workflows.

## Figures and Tables

**Figure 1:**
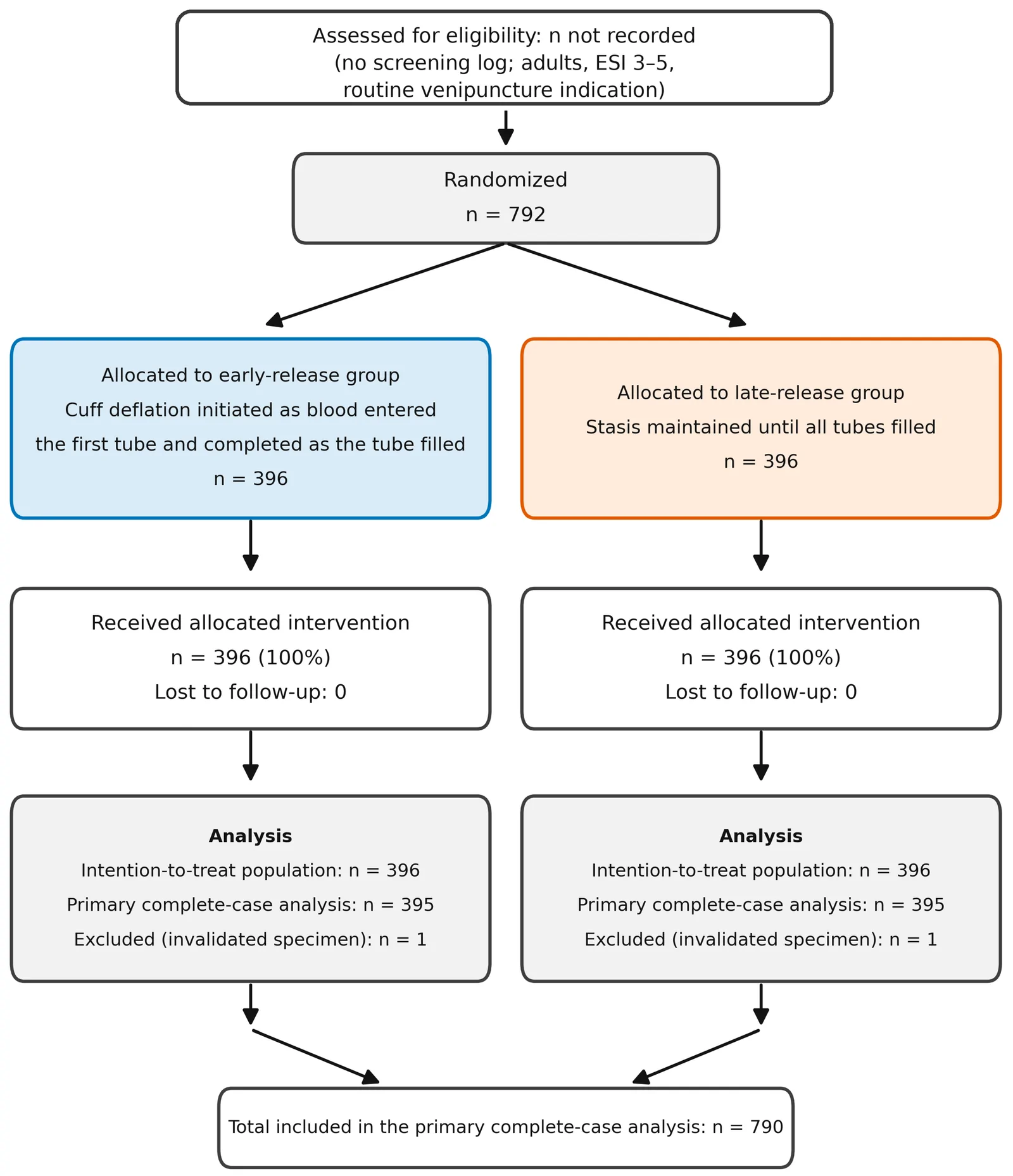
CONSORT participant flow diagram. A screening log was not maintained, so the number of patients assessed for eligibility and the number excluded were not recorded; the eligibility criteria are given in Materials and Methods. No patient who was approached declined to participate. Of 792 patients randomized, 396 were allocated to early release (cuff deflation initiated as blood entered the first tube and completed as the tube filled) and 396 to late release (stasis maintained until all tubes filled); all received the allocated intervention, and none were lost to follow-up. All 792 randomized patients formed the intention-to-treat population; the primary complete-case analysis included 790 patients (395 per group), with 1 specimen invalidated by the laboratory in each group (Patient 569 in early release; Patient 394 in late release; Supplementary Table S4).

**Figure 2:**
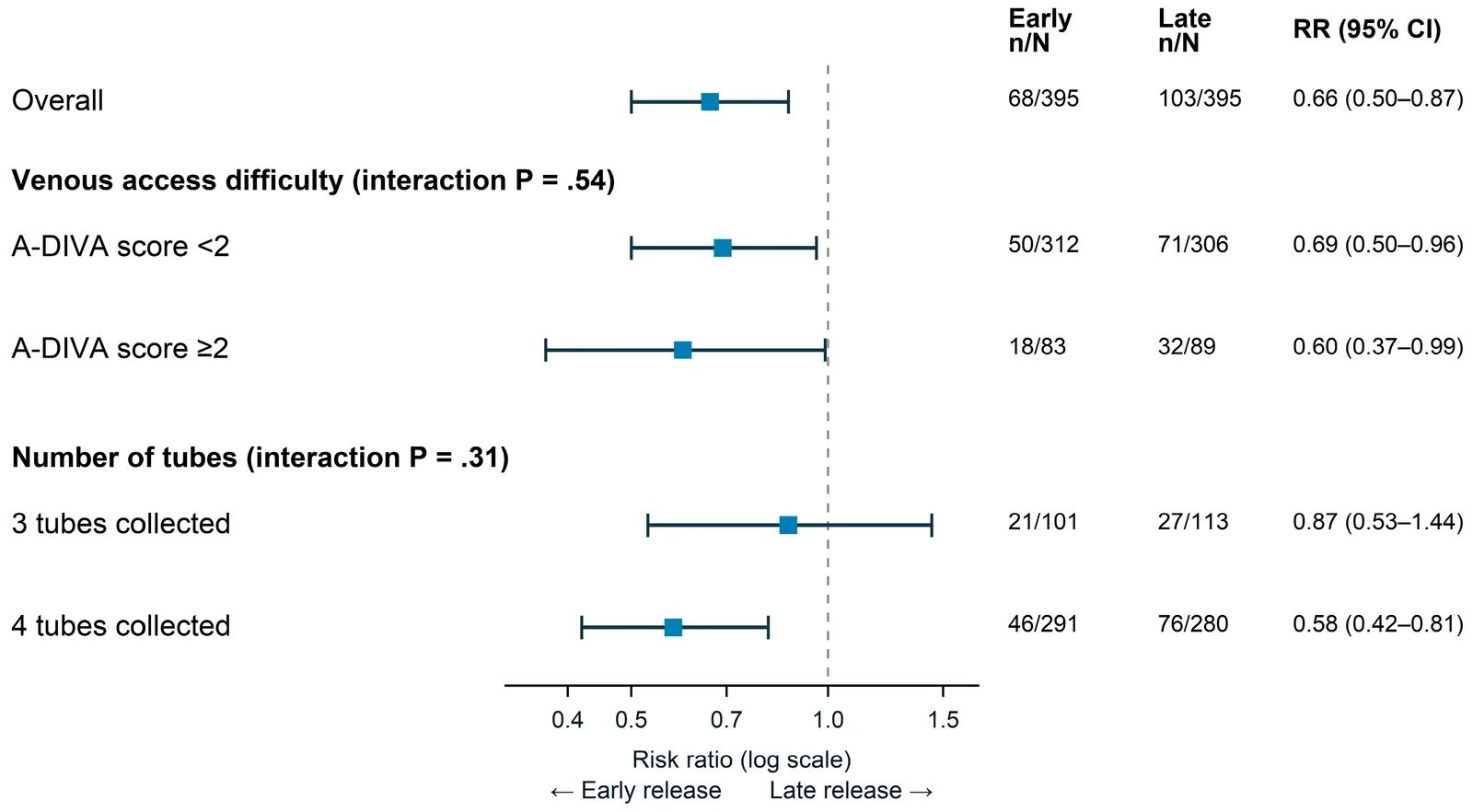
Forest plot of the primary outcome overall and within prespecified subgroups defined by venous access difficulty (A-DIVA score of 2 or greater versus below 2) and number of tubes collected. Relative risks are shown with 95% CIs on a logarithmic scale. No statistically significant interaction was detected (*p* = 0.54 for venous access difficulty and *p* = 0.31 for tube count). The 5-tube stratum (6 patients randomized; 5 in the primary analysis population) was too small to estimate an effect and was therefore not plotted.

**Table 1:** Baseline demographic, clinical, and procedural characteristics.

Characteristic	Early-Release Group (n = 396)	Late-Release Group (n = 396)
**Demographic**		
Age, y—median (IQR)	45.0 (33.0–58.0)	45.0 (32.0–57.0)
Female—n (%)	220 (55.6)	233 (58.8)
Height, cm—mean ± SD	167.2 ± 8.4	168.2 ± 8.4
Weight, kg—median (IQR)	74.0 (64.0–85.2)	75.0 (65.0–86.0)
BMI, kg/m^2^—median (IQR)	26.4 (22.9–30.1)	26.4 (22.9–29.9)
**Triage**		
Yellow (ESI 3)—n (%)	122 (30.8)	108 (27.3)
Green (ESI 4–5)—n (%)	274 (69.2)	288 (72.7)
**Comorbidity**		
Hypertension—n (%)	74 (18.7)	81 (20.5)
Diabetes mellitus—n (%)	59 (14.9)	51 (12.9)
Coronary artery disease—n (%)	27 (6.8)	25 (6.3)
Other (COPD, CKD, thyroid, asthma, HL)—n (%)	46 (11.6)	53 (13.4)
≥1 comorbidity—n (%)	139 (35.1)	134 (33.8)
≥2 comorbidities—n (%)	55 (13.9)	57 (14.4)
**Venous access difficulty (modified A-DIVA)**		
A-DIVA total score—median (IQR)	0.0 (0.0–1.0)	0.0 (0.0–1.0)
A-DIVA ≥ 2 (moderate/high predicted-risk categories)—n (%)	84 (21.2)	90 (22.7)
Previous difficult cannulation—n (%)	56 (14.1)	55 (13.9)
Operator-anticipated difficulty—n (%)	49 (12.4)	67 (16.9)
No palpable vein—n (%)	59 (14.9)	72 (18.2)
No visible vein—n (%)	73 (18.4)	72 (18.2)
Vein diameter < 3 mm—n (%)	91 (23.0)	80 (20.2)
**Procedure**		
Nurse experience, y—median (IQR)	5.0 (3.0–8.0)	5.0 (3.0–12.0)
3 tubes—n (%)	101 (25.5)	113 (28.5)
4 tubes—n (%)	292 (73.7)	280 (70.7)
5 tubes—n (%)	3 (0.8)	3 (0.8)
Antecubital site—n (%)	371 (93.7)	364 (91.9)
Dorsum of hand—n (%)	25 (6.3)	32 (8.1)
Successful on first attempt—n (%)	346 (87.4)	348 (87.9)
Number of attempts—median (IQR)	1.0 (1.0–1.0)	1.0 (1.0–1.0)

Note. Data are median (interquartile range [IQR]), mean ± standard deviation [SD], or n (%). Because this was a randomized trial, hypothesis testing for baseline differences was not performed; imbalances were addressed in the adjusted model. Comorbidities with prevalence < 5% are combined in a single row. A-DIVA = modified Adult Difficult Intravenous Access scale (scores ≥ 2 comprise the moderate- and high-predicted-risk categories); BMI = body mass index; CKD = chronic kidney disease; COPD = chronic obstructive pulmonary disease; ESI = Emergency Severity Index; HL = hyperlipidemia.

**Table 2:** Primary endpoint: proportion of samples with a hemolysis index of 1+ or greater, and effect measures.

Outcome	Early Release	Late Release	Effect (95% CI)
Hemolysis (HI ≥ 1+), n/N (%)	68/395 (17.2)	103/395 (26.1)	—
Absolute risk reduction	—	—	8.86% (3.12–14.54)
Risk ratio	—	—	0.660 (0.503–0.867)
Relative risk reduction	—	—	34.0%
Number needed to treat	—	—	11.3 (6.9–32.1)
Unadjusted odds ratio	—	—	0.590 (0.418–0.832)
Adjusted odds ratio *	—	—	0.580 (0.409–0.822)

Note. The primary comparison was a complete-case analysis of the 790 patients with a valid hemolysis index (395 per group), analyzed according to randomized allocation; the intention-to-treat population comprised all 792 randomized patients. *p* = 0.003 for the risk difference, the risk ratio, and the unadjusted odds ratio, and *p* = 0.002 for the adjusted odds ratio, from the continuity-corrected Pearson chi-square test for the risk difference and from Wald tests on the log scale for the ratio measures. The confidence interval for the risk difference was calculated with the Newcombe square-and-add Wilson method, and that for the risk ratio with the Katz logarithmic method. The interval for the unadjusted odds ratio was calculated with the Woolf (logit) method, that for the adjusted odds ratio with the Wald interval derived from the fitted logistic-regression coefficient, and that for the number needed to treat from the inverse of the risk-difference confidence interval. * Adjusted for total A-DIVA score, triage category, and number of venipuncture attempts (prespecified model). An extended 7-covariate sensitivity model (age, sex, body mass index, nurse experience, number of tubes collected, total A-DIVA score, and triage category; number of venipuncture attempts not included) gave an adjusted odds ratio of 0.587 (95% CI, 0.413–0.834; Supplementary Table S1). CI = confidence interval; HI = hemolysis index; NNT = number needed to treat, the number of patients managed with early release required to prevent one additional laboratory-defined hemolyzed specimen (HI ≥ 1+) under the study conditions.

## Data Availability

De-identified participant data will be available from the corresponding author upon reasonable request after publication, subject to institutional ethics approval and applicable data protection requirements.

## Supplementary Materials

The following supporting information can be downloaded at https://www.mdpi.com/article/10.3390/medicina62102009/s1, Table S1, Adjusted logistic-regression model for the primary outcome; Table S2, Missing-data sensitivity analyses; Supplementary Text S3, Descriptive allocation checks and exploratory analyses; and Table S4, Patient-level account of the 12 laboratory-rejected specimens and their reconciliation with primary-outcome evaluability. The Statistical Analysis Plan (Version 1.1, locked 30 May 2026, and Version 1.2) is provided as Supplementary Materials. A consolidated Study Protocol (Version 1.0, 8 October 2026), which also describes how the final procedures relate to the initial ethics application, is provided as Supplementary Materials. The completed CONSORT 2025 checklist is provided as non-published submission material.
