# Supplementary material for: Effect of Early Tourniquet Release on Hemolysis in Emergency Department Phlebotomy: The TOURNI-ED Randomized Trial
Source: Medicina (Kaunas). 2026 Oct 17;62(10):2009. doi: 10.3390/medicina62102009 (PMC13707226; doi:10.3390/medicina62102009)
Supplement: Supplementary file 1 [file medicina-62-02009-s001.zip › CONSORT_2025_checklist_TOURNI-ED.pdf]

CONSORT 2025 Checklist — TOURNI-ED Trial

Checklist of information to include when reporting a randomised trial (Hopewell S, Chan AW, Collins GS, et al. CONSORT 2025 statement: updated guideline for reporting randomised trials. BMJ. 2025;389:e081123). Section references refer to the revised manuscript (TOURNI_ED_Revised_Clean); the manuscript is not typeset, so locations are given by section rather than page number.

**Section/topic**	**Item No**	**Checklist item**	**Reported in manuscript (section)**
Title and abstract	1a	Identification as a randomised trial	Title: "…The TOURNI-ED Randomized Trial"
	1b	Structured summary of the trial design, methods, results, and conclusions	Abstract (structured: Background and Objectives, Materials and Methods, Results, Conclusions)
Open science	2	Name of trial registry, identifying number (with URL) and date of registration	Materials and Methods, Design and setting (ClinicalTrials.gov NCT07617103, URL, submission and posting dates)
	3	Where the trial protocol and statistical analysis plan can be accessed	Materials and Methods, Design and setting; Supplementary Materials statement (SAP Versions 1.1 and 1.2 and consolidated protocol supplied with reviewer materials)
	4	Where and how the individual de-identified participant data, statistical code and any other materials can be accessed	Data Availability Statement
	5a	Sources of funding and other support, and role of funders	Funding ("no external funding"); sponsor role stated in Design and setting
	5b	Financial and other conflicts of interest of the manuscript authors	Conflicts of Interest
Introduction	6	Scientific background and rationale	Introduction
	7	Specific objectives related to benefits and harms	Introduction (final paragraph); harms addressed via prespecified composite complication outcome (Materials and Methods, Outcomes)
Methods	8	Details of patient or public involvement in the design, conduct and reporting of the trial	Materials and Methods, Patient and public involvement (none; dissemination plans stated)
	9	Description of trial design including type of trial, allocation ratio, and framework	Materials and Methods, Design and setting (single-center, parallel-group, 1:1, superiority)

	10	Important changes to the trial after it commenced including any outcomes or analyses that were not prespecified, with reason	Design and setting (pre-enrollment refinements described; no post-enrollment changes to recruitment or intervention); Statistical analysis (supportive additional analyses identified; numerical HI scale and reporting categories distinguished); Outcomes (stasis endpoint reporting note)
	11	Settings and locations where the trial was conducted	Design and setting (emergency department of a tertiary teaching hospital, Marmara University Pendik Training and Research Hospital)
	12a	Eligibility criteria for participants	Materials and Methods, Participants
	12b	If applicable, eligibility criteria for sites and for individuals delivering the interventions	Materials and Methods, Participants (final paragraph) (venipuncture by emergency department nurses for whom phlebotomy is a routine duty; standardized briefing before enrollment)
	13	Intervention and comparator with sufficient details to allow replication	Materials and Methods, Intervention (cuff pressure 60 mm Hg, device, needle, site, tube order, release timings)
	14	Prespecified primary and secondary outcomes, including measurement variable, analysis metric, method of aggregation, and time point	Materials and Methods, Outcomes (HI ≥1+ proportion on the serum separator gel tube; ordinal HI distribution, repeat venipuncture, stasis duration, composite complication; exploratory outcomes)
	15	How harms were defined and assessed	Materials and Methods, Outcomes (composite definition; events assessed by the nurse during the procedure and for 30 minutes afterwards, recorded by type)
	16a	How sample size was determined, including all assumptions	Materials and Methods, Sample size (assumed rates 12% vs. 6%, alpha .05, 80% power, attrition allowance)
	16b	Explanation of any interim analyses and stopping guidelines	Design and setting ("there were no interim analyses and no stopping rules")
	17a	Who generated the random allocation sequence and the method	Materials and Methods, Randomization (sequence generated before

		used	enrollment by the principal investigator, who did not enroll participants or perform venipuncture, with Research Randomizer, randomizer.org)
	17b	Type of randomisation and details of any restriction	Randomization (1:1 restricted allocation; 198 permuted blocks of size 4, no stratification; block size concealed); Supplementary Text S3
	18	Mechanism used to implement the random allocation sequence, describing steps to conceal the sequence until interventions were assigned	Randomization (sequence embedded in the electronic data collection application, not accessible to the enrolling nurse, released only after eligibility confirmation; no sealed envelopes)
	19	Whether the personnel who enrolled and those who assigned participants to the interventions had access to the random allocation sequence	Randomization (application-released allocation after enrollment; enrolling nurse had no access)
	20a	Who was blinded after assignment to interventions	Randomization (participants and nurse not blinded; outcome assessor blinded; analyst working on a neutrally coded dataset, unaware of group identity)
	20b	If blinded, how blinding was achieved and description of the similarity of interventions	Randomization (LIS barcode identification; allocated group appeared neither on the request nor on the tube); blinding of participants/care providers not applicable (impossible by design, stated)
	21a	Statistical methods used to compare groups for primary and secondary outcomes, including harms	Materials and Methods, Statistical analysis (Newcombe/Katz intervals, chi-square tests, Mann-Whitney U, logistic regression, Bonferroni alpha .0125)
	21b	Definition of who is included in each analysis, and in which group	Statistical analysis and Results (ITT = all 792 randomized; primary complete-case n = 790, analyzed as randomized)
	21c	How missing data were handled in the analysis	Statistical analysis (complete-case primary; all four assignments of the 2 missing outcomes examined); Supplementary Table S2
	21d	Methods for any additional analyses,	Statistical analysis (supportive analyses

		distinguishing prespecified from post hoc	explicitly listed as beyond the plan); Results, Subgroup analyses; Supplementary Text S3
Results	22a	For each group, numbers of participants randomly assigned, receiving intended intervention, and analysed for the primary outcome	Results (first paragraph); Figure 1 (CONSORT flow diagram)
	22b	For each group, losses and exclusions after randomisation, together with reasons	Results (no losses; 1 invalidated specimen per group with reasons: barcode mismatch, insufficient serum volume); Figure 1; Table S4
	23a	Dates defining the periods of recruitment and follow-up	Design and setting (enrollment June 1–July 30, 2026; follow-up ended with the laboratory result of the index sample)
	23b	If relevant, why the trial ended or was stopped	Not applicable — the trial was completed as planned (no interim analyses or stopping rules; Design and setting)
	24a	Intervention and comparator as they were actually administered (who delivered, adherence, fidelity)	Results, Duration of venous stasis and protocol adherence (measured stasis as fidelity indicator; no deviation from allocated release timing); Intervention was delivered by ED nurses (Methods)
	24b	Concomitant care received during the trial for each group	Intervention (“No concomitant care was standardized or restricted by the protocol; all patients received routine emergency department care”)
	25	A table showing baseline demographic and clinical characteristics for each group	Table 1
	26	For each primary and secondary outcome, by group: numbers analysed, numbers with available data, results, effect size and precision; absolute and relative effects for binary outcomes	Results; Table 2 (primary outcome: ARR, RR, OR, NNT with 95% CIs; 395/395 evaluable per group); Table 3 (secondary/exploratory outcomes; denominators footnoted)
	27	All harms or unintended events in each group	Table 3 (composite complication outcome with components by group); Results, Table 3 (composite complication outcome with components by group); Discussion, Safety

	28	Any other analyses performed, including subgroup and sensitivity analyses, distinguishing pre-specified from post hoc	Results, Subgroup analyses (Figure 2); missing-data sensitivity analyses (Table S2); Supplementary Text S3; minimum detectable difference given the observed control rate (Sample size)
Discussion	29	Interpretation consistent with results, balancing benefits and harms, and considering other relevant evidence	Discussion (including Safety and Nature of the outcome paragraphs)
	30	Trial limitations, addressing sources of potential bias, imprecision, generalisability, and, if relevant, multiplicity of analyses	Limitations section (single center, outcome threshold, screening log absence, unblinded ascertainment, fixed cuff pressure, multiplicity addressed via Bonferroni and exploratory labelling)
