# Supplementary material for: Effect of Early Tourniquet Release on Hemolysis in Emergency Department Phlebotomy: The TOURNI-ED Randomized Trial
Source: Medicina (Kaunas). 2026 Oct 17;62(10):2009. doi: 10.3390/medicina62102009 (PMC13707226; doi:10.3390/medicina62102009)
Supplement: Supplementary file 1 [file medicina-62-02009-s001.zip › Statistical_Analysis_Plan_TOURNI-ED_v1.1.pdf]

STATISTICAL ANALYSIS PLAN

Effect of Early Tourniquet Release on Hemolysis in Emergency Department Phlebotomy: The TOURNI-ED Randomized Trial

Study Administrative Item	Trial Specification
Official Study Title	Effect of Early Tourniquet Release on Hemolysis in Emergency Department Phlebotomy: A Superiority Randomized Controlled Trial (TOURNI-ED)
Protocol ID / Ethics Registration	09.2026.829 (Marmara University Faculty of Medicine Clinical Research Ethics Committee; Approval Date: 22 May 2026)
ClinicalTrials.gov Identifier	NCT07617103 (Submitted 23 May 2026, Registered Prior to Enrollment)
Trial Registry PRS ID	S000H78F00000059
Principal Investigator	Emir Ünal, MD, Department of Emergency Medicine, Marmara University Pendik Training and Research Hospital
Document Version & Status	Version 1.1
Document Lock Date	30 May 2026 (Prior to trial enrollment launch on 1 June 2026)

1. Purpose, Scope, and Document Governance

This Statistical Analysis Plan (SAP) prospectively specifies the statistical principles, endpoint definitions, population structures, hypothesis testing procedures, and reporting frameworks for the TOURNI-ED trial. The document has been developed in strict accordance with the International Council for Harmonisation (ICH) E9 guidance on Statistical Principles for Clinical Trials and the CONSORT 2025 Statement.

This plan is governed alongside the institutional ethics-approved study protocol (Protocol No: 09.2026.829) and the ClinicalTrials.gov registration (NCT07617103). It provides the complete prespecified analytical blueprint for the study prior to patient recruitment.

Document Lock and Integrity: The governance and document control rules for this trial are established as follows:

- • Formal Prespecification: This document is formally signed, dated, and locked prior to the randomization of the first study participant.

- • Tamper-Proof Archiving: The signed version is preserved as a read-only document with a cryptographic SHA-256 checksum and deposited in the institutional clinical research archive.
- • Handling of Protocol Amendments: Any amendment to this analysis plan after enrollment begins shall require a formal written protocol addendum documenting the rationale, the exact modification, and confirmation that treatment allocation codes remain masked.
- • Alignment with Trial Registry: The primary and secondary endpoints described herein are concordant with the entries registered on ClinicalTrials.gov (NCT07617103).

Document Version	Effective Date	Document Status & Scope
Version 1.0	30 May 2026	Initial formal version prior to enrollment.
Version 1.1	30 May 2026	Final prespecified plan incorporating verified permuted block structure (198 blocks of 4) and strict allocation concealment parameters.

2. Study Design and Administrative Architecture

The TOURNI-ED trial is a single-center, parallel-group, superiority randomized controlled trial conducted at the Emergency Department of Marmara University Pendik Training and Research Hospital, Istanbul, Turkey. The trial evaluates whether early release of sphygmomanometer-applied venous stasis reduces phlebotomy-induced hemolysis compared with usual care late release.

Design Element	Operational Specification
Trial Architecture	Single-center, parallel-group, superiority randomized controlled trial (1:1 allocation)
Study Setting	Adult Emergency Department, Marmara University Pendik Training and Research Hospital
Target Population	Adult patients (≥18 years) triaged to green or yellow acuity areas requiring routine venous blood collection (≥2 collection tubes)
Experimental Intervention (Group A)	Early tourniquet release: Sphygmomanometer cuff inflated to 60 mmHg; cuff released (deflated to 0 mmHg) immediately upon observing blood flow into the first tube (sodium citrate, 2.7 mL, blue cap). All subsequent tubes collected without active stasis.

Design Element	Operational Specification
Active Comparator / Control (Group B)	Late tourniquet release (Usual Care): Sphygmomanometer cuff inflated to 60 mmHg; stasis maintained continuously throughout the collection sequence and released only after the final tube completes filling.
Standardized Order of Draw	Standardized CLSI H03-A6 sequence for all patients: (1) Sodium citrate (blue cap), (2) Serum separator gel tube (SST, yellow cap; primary outcome tube), (3) K2-EDTA (purple cap), followed by any routine clinical add-on tubes.
Target Sample Size	792 randomized participants (396 per study arm)
Primary Endpoint	Laboratory-confirmed hemolysis in the serum separator gel tube (Hemolysis Index ≥ 1+), assessed by the automated chemistry analyzer.
Interim Analysis & Stopping	No interim efficacy analysis or formal stopping boundaries are planned. Trial recruitment will continue to the predetermined target.

3. Randomization, Allocation Concealment, and Masking

3.1 Randomization Sequence Generation

A 1:1 restricted allocation sequence of 792 assignments (396 per arm) was generated prior to trial initiation using an electronic pseudorandom number generator (Research Randomizer, randomizer.org) without stratification. The sequence was structured as 198 permuted blocks of size four, with each consecutive block containing two allocations to Group A and two to Group B. To prevent selection bias and maintain strict unpredictability, the block size was not disclosed to the clinical, nursing, or enrollment personnel. The master randomization schedule is archived in a password-protected, encrypted directory accessible solely to the independent randomizer.

3.2 Allocation Concealment Mechanism

Allocation concealment was maintained using a centralized electronic data collection application at the bedside. Enrolling nurses and research staff had no access to the sequence or upcoming assignments. A participant’s group assignment was revealed sequentially and only after written informed consent was verified, all eligibility criteria were confirmed, and the participant was formally registered in the electronic trial log. Sealed paper envelopes were strictly prohibited to prevent deciphering or subversion of the allocation sequence.

3.3 Masking and Blinding Safeguards

Masking Strategy: Due to the nature of the procedural intervention (cuff deflation timing at the bedside), participating patients and phlebotomy nurses cannot be blinded to treatment allocation. However, comprehensive masking is maintained across all downstream evaluators:

- • Laboratory Outcome Assessors: Laboratory technicians and automated clinical chemistry analyzers (Beckman Coulter DxC series) are fully blinded to treatment allocation. Each blood tube is identified solely by an automated laboratory information system (LIS) barcode containing no group indicators.
- • Trial Biostatistician: The trial statistician will conduct primary and supportive analyses on a masked dataset where treatment groups are designated by neutral alphanumeric codes (e.g., Group X vs. Group Y). Decoding will take place only after database lock and formal execution of the prespecified code.

4. Analysis Populations

Population	Prespecified Definition and Analytic Purpose
Intention-to-Treat (ITT) Population	Consists of all randomized participants, analyzed according to their randomly assigned treatment group, regardless of procedural adherence or deviations. Represents the primary administrative population.
Primary Outcome (Complete-Case ITT) Population	All randomized ITT participants who have a valid, evaluable hemolysis index recorded for the primary serum separator gel tube. Forms the primary population for the confirmatory hypothesis test.
Per-Protocol (PP) Population	Subgroup of the ITT population who completed blood collection without major procedural protocol deviations. Major deviations are defined a priori as: (1) Cuff release timing violating the allocated protocol, or (2) Phlebotomy draw order deviating from the standard CLSI H03-A6 sequence. Serves as a sensitivity population.
Safety Population	Consists of all randomized participants in whom venipuncture was initiated, analyzed according to the actual procedure received. Utilized for adverse event and procedural complication summaries.

5. General Statistical Principles

All statistical hypothesis tests will be conducted as two-sided tests at the nominal significance level of alpha = 0.05, unless otherwise adjusted for multiplicity.

- • Continuous Variables: Data will be evaluated for distributional normality using visual inspection of Q-Q plots, histograms, and the Shapiro-Wilk test. Approximately symmetric continuous variables will be reported as mean with standard deviation (SD). Variables exhibiting marked skewness will be summarized as median with interquartile range (IQR; 25th to 75th percentiles).
- • Categorical Variables: Binary and polytomous categorical variables will be presented as absolute frequencies (counts) and relative percentages (n, %), with the explicit denominator clearly stated.
- • Baseline Characteristics: Baseline clinical, demographic, and phlebotomy parameters will be summarized by treatment arm. In accordance with CONSORT 2025 guidelines, significance tests of baseline differences will not be performed, as any observed differences reflect chance in a randomized trial.
- • Unit of Analysis: The individual patient undergoing a single phlebotomy episode is the independent unit of analysis.

6. Study Variables and Operational Data Handling

6.1 Hemolysis Index (HI) Measurement

Hemolysis is quantified in the clinical laboratory using the automated Beckman Coulter DxC clinical chemistry analyzer. The analyzer optically assesses free hemoglobin in the serum separator gel tube (SST, yellow cap) through spectrophotometric bichromatic absorbance measurements at 570/600 nm.

The analyzer maps free hemoglobin to an ordinal scale from 0 to 5+, defined as: 0 (negative, <50 mg/dL), 1+ (50–99 mg/dL), 2+ (100–199 mg/dL), 3+ (200–299 mg/dL), 4+ (300–500 mg/dL), and 5+ (>500 mg/dL). For the primary endpoint, a binary indicator will be derived where an index of 1+ or greater is classified as hemolyzed, and 0 is classified as non-hemolyzed.

6.2 Procedural Timings and Covariates

- • Duration of Venous Stasis: Measured in seconds using a digital stopwatch, defined as the interval from the start of sphygmomanometer cuff inflation (60 mmHg) until complete cuff deflation (0 mmHg).
- • Modified A-DIVA Score: Assessed prior to puncture on a 0–5 scale based on five clinical criteria: history of difficult access, non-palpable vein, non-visible vein, vein diameter <3 mm, and operator-anticipated difficulty. Scores ≥2 are classified as difficult venous access.
- • Number of Venipuncture Attempts: Recorded as the total number of skin punctures required to achieve successful blood collection.
- • Triage Acuity Category: Classified at emergency triage as Green (semi-urgent) or Yellow (urgent) according to institutional triage standards.

6.3 Laboratory Specimen Rejection Rules

Handling of rejected laboratory specimens is prespecified as follows:

- • Non-Hemolysis Laboratory Rejection: If the primary SST tube is rejected by the central laboratory due to preanalytical errors unrelated to hemolysis (e.g., insufficient blood volume, specimen

clotting, tube breakage), the primary outcome is missing. These participants are excluded from the complete-case primary analysis and evaluated in missing-data sensitivity scenarios.

- • Hemolysis-Induced Rejection: If an SST tube is rejected or deemed unprocessable specifically due to gross hemolysis, it is classified as hemolyzed (HI ≥ 1+).
- • Rejection in Non-Primary Tubes: Rejection of non-SST tubes (e.g., coagulation tube clotting) will be documented in procedural logs but does not affect primary endpoint evaluation.

7. Sample Size and Statistical Power

The study is sized to detect a clinically meaningful superiority of early tourniquet release over late release in reducing phlebotomy-induced hemolysis. Based on published emergency department phlebotomy literature, the baseline hemolysis rate under standard care (late release) is estimated at 12%. Early tourniquet release is hypothesized to achieve an absolute reduction of 6%, yielding an anticipated hemolysis rate of 6% in the experimental arm.

Sample size determination parameters:

- • Assumed Proportion in Group B (Late Release / Control): p2 = 0.12 (12.0%)
- • Assumed Proportion in Group A (Early Release / Intervention): p1 = 0.06 (6.0%)
- • Target Effect Size: Absolute Risk Difference of -6.0% (Relative Risk = 0.50)
- • Significance Level (Alpha): Two-sided alpha = 0.05
- • Statistical Power (1 - Beta): 80% (beta = 0.20)
- • Test Method: Two-tailed normal approximation for two independent proportions with pooled variance.

Under these specifications, a minimum of 356 evaluable participants per arm (712 total) is required. To accommodate an anticipated ~10% rate of uninterpretable, lost, or rejected specimens, the recruitment target is set to 396 participants per arm, resulting in a total planned enrollment of 792 participants.

8. Primary Endpoint Statistical Analysis

8.1 Primary Hypothesis Test

The primary hypothesis states that early tourniquet release reduces the proportion of hemolyzed serum separator tube samples compared with late tourniquet release:

H0: p(Early) = p(Late) versus H1: p(Early) != p(Late)

The primary comparison will be performed in the complete-case ITT population using the Pearson chi-square test with Yates continuity correction at a two-sided alpha level of 0.05.

8.2 Effect Measures and Precision Estimation

Treatment effects will be quantified through three complementary metrics:

- **1.** Absolute Risk Difference (RD): Calculated as p(Early) - p(Late), presented with a two-sided 95% confidence interval computed via the Newcombe square-and-add method based on Wilson score intervals.

- **2.** Relative Risk (RR): Calculated as p(Early) / p(Late), presented with a 95% confidence interval computed via the Katz logarithmic method.
- **3.** Number Needed to Treat (NNT): Calculated as the reciprocal of the absolute risk difference (1 / |RD|), rounded to one decimal place, with confidence bounds obtained by inverting the risk difference confidence limits.

8.3 Supportive Multivariable Adjusted Analysis

To account for baseline prognostic characteristics, a supportive multivariable logistic regression model will be fitted. The dependent variable is primary hemolysis status (1 = hemolyzed, 0 = non-hemolyzed), and the primary explanatory variable is treatment assignment (Group A vs. Group B).

The model will adjust simultaneously for three prespecified baseline covariates without stepwise selection:

- • Modified A-DIVA Total Score (continuous integer score, 0 to 5);
- • Emergency Triage Acuity (binary: Yellow urgent vs. Green semi-urgent); and
- • Number of Venipuncture Attempts (continuous integer count).

Results will be expressed as adjusted Odds Ratios (aOR) with 95% Wald confidence intervals. The unadjusted chi-square test remains the primary basis for statistical inference; the adjusted model serves as supportive corroboration.

9. Secondary Endpoints Analysis

Secondary outcomes provide comprehensive evaluation of hemolysis severity, clinical operational consequences, procedural mechanics, and patient safety.

Secondary Endpoint	Metric & Operationalization	Statistical Hypothesis Test	Significance Level
Ordinal Hemolysis Index	Six-level ordinal categorization of hemolysis severity (0, 1+, 2+, 3+, 4+, 5+)	Mann-Whitney U test (Wilcoxon rank-sum test)	Bonferroni alpha = 0.0125
Repeat Phlebotomy Rate	Proportion of participants requiring a repeat blood draw secondary to laboratory hemolysis	Pearson chi-square test or Fisher's exact test (if expected cell < 5)	Bonferroni alpha = 0.0125
Venous Stasis Duration	Interval from cuff inflation to complete deflation (seconds); also proportion >60 seconds	Mann-Whitney U test (continuous); Pearson chi-square (>60 s proportion)	Bonferroni alpha = 0.0125
Composite Complication Rate	Occurrence of ≥1 procedural complication (hematoma, swelling, oozing, vasovagal reaction, nerve injury, arterial puncture, or >2 attempts)	Pearson chi-square test (without continuity correction) or Fisher's exact test	Bonferroni alpha = 0.0125

10. Multiplicity and Type I Error Control

Type I error control is structured through a prespecified hierarchical framework:

- **1. Primary Testing:** Primary Endpoint: Evaluated at a dedicated two-sided alpha level of 0.05. Statistical significance of the primary endpoint establishes the overall efficacy of the intervention.
- **2. Secondary Testing:** Confirmatory Secondary Endpoints: To control the family-wise error rate across the four key secondary endpoints, a Bonferroni adjustment will be applied (overall alpha of 0.05 / 4 = 0.0125). A secondary endpoint will be declared statistically significant if its two-sided p-value is < 0.0125.
- **3. Exploratory Analyses:** Exploratory and Subgroup Analyses: All additional exploratory endpoints, post-hoc examinations, and subgroup comparisons will be reported with nominal p-values and unadjusted 95% confidence intervals, and will be interpreted strictly as hypothesis-generating.

11. Prespecified Subgroup Analyses

To explore treatment effect consistency across key patient subgroups, two prespecified subgroup analyses of the primary endpoint will be performed:

- **1.** Venous Access Difficulty: Patients with a modified A-DIVA score < 2 (low difficulty) versus modified A-DIVA score ≥ 2 (high difficulty).

- **2.** Phlebotomy Tube Burden: Stratification by the total number of collection tubes drawn (3 tubes vs. 4 tubes vs. 5 tubes), evaluating the interaction between stasis duration and tube burden.

Subgroup effects will be estimated within each stratum as Relative Risks and Absolute Risk Differences with 95% confidence intervals. Formal assessment of effect modification will be conducted by incorporating a treatment-by-subgroup interaction term in a multivariable logistic regression model. As the trial is not powered to detect subtle interactions, subgroup findings will be interpreted with appropriate caution.

12. Missing Data Handling and Sensitivity Imputation

The primary analysis will be conducted on complete cases within the ITT population. To evaluate the robustness of the primary findings against missing outcome data (due to rejected or unprocessable tubes), four prespecified sensitivity scenarios will be executed:

- **Scenario 1 (Benchmark):** Complete-Case Scenario: Primary benchmark excluding participants with missing hemolysis data.
- **Scenario 2 (Best-Case):** Best-Case Imputation: All missing primary outcomes in both study arms are imputed as non-hemolyzed (HI = 0).
- **Scenario 3 (Worst-Case):** Worst-Case Imputation: All missing primary outcomes in both study arms are imputed as hemolyzed (HI ≥ 1+).
- **Scenario 4 (Tipping-Point):** Tipping-Point Imputation: Extreme conservative scenario where missing outcomes in the early release arm are imputed as hemolyzed (HI ≥ 1+) and missing outcomes in the late release arm are imputed as non-hemolyzed (HI = 0).

The range of risk differences and corresponding confidence bounds across all four scenarios will be reported. Missing covariate values in multivariable modeling will be documented; complete-case covariate analysis will be used without multiple imputation.

13. Sensitivity Analyses

The stability and validity of the primary trial outcome will be evaluated through three prespecified sensitivity analyses:

- **1.** Per-Protocol Analysis: Re-estimation of the primary endpoint comparison in the Per-Protocol population (excluding major procedural deviations).
- **2.** Covariate-Adjusted Analysis: Direct comparison of the multivariable adjusted odds ratio against the unadjusted primary effect estimate.
- **3.** Missing Data Scenarios: Comparison of effect estimates across the four imputation scenarios detailed in Section 12.

14. Safety and Complications Evaluation

Procedural safety will be evaluated in the Safety Population. Complications and adverse events will be tabulated by study group as:

- • Participant-level complication incidence: Number and percentage of participants experiencing at least one adverse event;
- • Event-level frequencies: Counts of specific complication types, including localized hematoma, insertion site oozing, local swelling, vasovagal syncope or presyncope, suspected nerve irritation, accidental arterial puncture, and repeated venipuncture attempts (>2 attempts).

Between-group differences in composite complication rates will be evaluated using Pearson's chi-square test without continuity correction (or Fisher's exact test if any expected cell frequency is below 5).

15. Statistical Software, Reproducibility, and Verification

Statistical analyses will be executed using Jamovi (current stable release) and verified in R (Version 4.3 or higher). Effect measures not natively calculated in jamovi (Newcombe Wilson score intervals for risk differences, Katz log intervals for relative risks, and exact NNT intervals) will be computed using documented, archived R code.

To ensure analytical integrity, an independent biostatistician will perform parallel double-programming of the primary endpoint and key secondary outcomes prior to database unmasking.

16. Reporting Standards, Trial Registration, and Archiving

Trial reporting will strictly adhere to the CONSORT 2025 Statement for randomized controlled trials. The manuscript will include a complete CONSORT participant flow diagram delineating enrollment, allocation, intervention receipt, specimen evaluability, and final analysis inclusions.

Any post-hoc or exploratory analyses conducted after database unblinding that were not prespecified in this document will be explicitly designated as 'post-hoc' in published reports, accompanied by the scientific justification for their execution.

17. Formal Approvals and Document Lock

By signing below, the Principal Investigator and Lead Biostatistician confirm that this Statistical Analysis Plan represents the finalized, prespecified analytical strategy for the TOURNI-ED trial, established and locked prior to the enrollment of the first study participant.

Role & Responsibility	Investigator Name & Affiliation	Signature & Approval Date
Principal Investigator	Emir Ünal, MD<br>Department of Emergency Medicine<br>Marmara University Faculty of Medicine	______________________________<br><br>Date: 30 May 2026
Lead Trial Biostatistician	Trial Biostatistician / Co-Investigator<br>Department of Biostatistics<br>Marmara University Faculty of Medicine	______________________________<br><br>Date: 30 May 2026
