# Supplementary material for: Effect of Early Tourniquet Release on Hemolysis in Emergency Department Phlebotomy: The TOURNI-ED Randomized Trial
Source: Medicina (Kaunas). 2026 Oct 17;62(10):2009. doi: 10.3390/medicina62102009 (PMC13707226; doi:10.3390/medicina62102009)
Supplement: Supplementary file 1 [file medicina-62-02009-s001.zip › Study_Protocol_TOURNI-ED_Consolidated_2026-10-08.pdf]

TOURNI ED Study Protocol

Consolidated description of the final study plan

Effect of Early Tourniquet Release on Hemolysis in Emergency Department Phlebotomy: The TOURNI-ED Randomized Trial

Document version: 1.0 | Prepared 8 October 2026 for peer review

This document brings together the final study procedures described in the revised manuscript and Statistical Analysis Plan (SAP). The investigators confirm that the refinements to the initial ethics application were completed before the first participant was enrolled on 1 June 2026. This consolidated document was prepared for peer review on the date shown above; it is not presented as the original prerecruitment protocol or a contemporaneous amendment record. Section 16 explains the relationship between the initial application and the final plan.

1. Study identification and source documents

Study site: Marmara University Pendik Training and Research Hospital, Emergency Department, Istanbul, Türkiye. Principal investigator: Emir Ünal, MD. Ethics reference reported in the manuscript: 09.2026.829. Trial registration: ClinicalTrials.gov NCT07617103.

The source documents are the Turkish clinical research application (ethics committee decision dated 22 May 2026, protocol code 09.2026.829), SAP Version 1.1 dated 30 May 2026, current SAP Version 1.2, and the revised manuscript. The original application is held in the study archive, and SAP Version 1.1 remains a separate document.

2. Rationale and objective

The study evaluates whether early release of standardized sphygmomanometer-induced venous stasis reduces laboratory-defined hemolysis compared with release after the collection tubes have filled. The intervention changes both the duration of stasis and whether subsequent tubes are collected under active stasis. The trial therefore assesses the combined procedural strategy; it does not isolate the independent effects of these components.

3. Design and setting

The study is a single-center, parallel-group, 1:1 randomized controlled superiority trial in a tertiary emergency department. The target sample is 792 participants, with 396 allocated to each arm. Enrollment took place from 1 June to 30 July 2026, as reported in the manuscript. Laboratory follow-up concerned the index collection; procedural adverse events were assessed during collection and for up to 30 minutes afterwards. There were no interim analyses or stopping rules.

4. Eligibility

Eligible participants are adults aged 18 years or older, triaged to Emergency Severity Index level 3 or levels 4–5, who require routine venipuncture and provide written informed consent. At least two collection tubes must have been ordered.

Exclusions are critical illness or hemodynamic instability; anticoagulant or thrombolytic therapy or circumstances in which collection of a coagulation tube is contraindicated; known hemolytic disease or active hemolysis; red blood cell transfusion within the preceding 24 hours; pregnancy at any gestational age; absence of a suitable peripheral vein; local pathology preventing antecubital collection; collection from an existing intravenous catheter; inability to consent; or declining participation. Each participant may be enrolled only once.

5. Recruitment and consent

Eligibility is assessed at the bedside before enrollment. Written informed consent is obtained before allocation is released. The consent information describes both cuff-release timings. Emergency department nurses perform venipuncture after a standardized pre-enrollment briefing. Routine emergency care is otherwise unrestricted.

A prospective screening log was not maintained. Accordingly, the total number assessed and exclusions by reason cannot be quantified from the study records; the participant flow diagram states this limitation.

6. Sequence generation and allocation concealment

The principal investigator, who did not enroll participants or perform venipuncture, generated the allocation schedule before enrollment using Research Randomizer (randomizer.org). The final schedule contains 792 assignments without stratification, arranged as 198 permuted blocks of four, with two assignments to each arm per block. The complete sequence was fixed before enrollment.

The sequence was embedded in the bedside electronic data collection application. Enrolling nurses could not access the schedule; allocation was released only after eligibility confirmation and enrollment. Block size was concealed from personnel responsible for enrollment and collection. No sealed envelopes were used in the implemented procedure. These statements describe the final method reported by the investigators; the properties of an ordered analysis dataset alone do not establish how the sequence was originally generated.

7. Blinding and identification of specimens

Participants and collecting nurses were not blinded because the consent information and visible cuff-release timing disclose the intervention. The laboratory technician recording the hemolysis index was blinded to allocation. The analyst used neutral group codes without knowing which represented early or late release. Sequence generation, collection, and analysis were assigned to different members of the team.

Each tube carried a separate laboratory information system barcode. The SST was identified by its barcode; allocated treatment did not appear on the tube or laboratory request. The hemolysis index associated with that specimen was entered into the study dataset.

8. Standard collection procedure

An identical aneroid sphygmomanometer (ERKA) was applied to the nondominant upper arm and inflated to 60 mm Hg. Blood was collected with a 21-gauge straight multisample needle and transparent holder; butterfly sets and intravenous catheters were not used. Collection was antecubital or, at the operator’s discretion, at another peripheral site, most often the dorsum of the hand.

The collection order was sodium citrate, 2.7 mL; serum separator gel (SST), 5 mL; and K2-EDTA, 3 mL. Additional tubes were ordered on clinical grounds before allocation was revealed. Their number was recorded. All other procedural elements were the same in both groups.

9. Allocated interventions

Early-release arm: cuff deflation was initiated when blood entered the first tube and was completed as that tube filled. Subsequent tubes were collected without active cuff-induced stasis.

Late-release arm: cuff pressure was maintained until the last collection tube filled, and the cuff was then released. The final definition refers to the last tube actually collected, including any clinically ordered additional tubes.

10. Hemolysis assessment and primary outcome

Hemolysis index (HI) was assessed on the primary SST specimen using the Beckman Coulter DxC 700 AU clinical chemistry analyzer. Laboratory consultation established before enrollment that the citrate and K2-EDTA tubes were processed on other analyzers and did not yield an HI; they were therefore not used for the hemolysis endpoint.

The primary outcome is the proportion of primary SST specimens with HI of 1+ or greater, corresponding to free hemoglobin of at least 50 mg/dL. The laboratory categories used for reporting are negative (<50 mg/dL), 1+ (50–99), 2+ (100–199), 3+ (200–299), 4+ (300–500), and the highest category (>500). The primary endpoint is binary. The ordinal endpoint concerns the ordered HI distribution; instrument or LIS numerical values should be distinguished from these clinical reporting labels.

11. Secondary outcomes and venous stasis duration

The final plan specifies four secondary outcomes: ordinal HI distribution; hemolysis-related repeat venipuncture; venous stasis duration; and a composite complication outcome.

Venous stasis duration is the interval from cuff inflation to complete cuff release. It differs from the initial application's total collection-time definition, which ended when the final tube filled. In the early-release arm, collection continues after cuff release. In the late-release arm, release occurs when the final tube fills, so the endpoints coincide operationally. The manuscript reports the measured variable as venous stasis duration, including the prespecified threshold of more than 60 seconds. The registry label "total blood collection time" and this operational definition are explicitly distinguished.

The complication composite includes any recorded hematoma or ecchymosis, local swelling, puncture-site oozing, vasovagal reaction, nerve injury, or arterial puncture, or more than two venipuncture attempts. Events are recorded by the collecting nurse during the procedure and for up to 30 minutes afterwards.

12. Exploratory outcomes and subgroup variables

Exploratory outcomes are serum potassium, lactate dehydrogenase, aspartate aminotransferase, and pain on a 0–10 visual analog scale. The final plan identifies venous access difficulty and collection-tube count as subgroup variables. The five-item modified Adult Difficult Intravenous Access score is recorded before venipuncture; the planned comparison is a score of at least two versus below two. Procedural variables include the number of attempts and the collecting nurse's years of experience. Operator identity was not recorded.

13. Sample size and analysis populations

The sample-size assumptions reported in the SAP and manuscript are a hemolysis rate of 12% with late release and 6% with early release, two-sided alpha of 0.05, and 80% power. The final recruitment target is 396 participants per arm, or 792 in total. The initial application specifies the same recruitment target; the explanatory sample-size calculation differs between that application and the manuscript. This consolidated document does not retrospectively replace the initial calculation.

All randomized participants constitute the intention-to-treat population. The primary comparison uses participants with an evaluable primary SST HI, retaining randomized allocation. Missing primary outcomes caused by specimen invalidation are excluded from the complete-case comparison and examined in sensitivity analyses. A restriction to participants adhering to the allocated intervention serves as a supportive per-protocol comparison where relevant.

14. Statistical analysis framework

The unadjusted primary comparison uses Pearson's chi-square test with Yates continuity correction. Risk difference, relative risk, and number needed to treat are reported with 95% confidence intervals. The SAP specifies Newcombe Wilson intervals for the risk difference and Katz logarithmic intervals for the risk ratio.

A supportive logistic regression adjusts for modified A-DIVA score, triage category, and number of venipuncture attempts. These covariates are also specified in the original ethics application. Continuous covariates enter as linear terms. The extended model with additional covariates is identified in the manuscript as a sensitivity analysis rather than as the prespecified supportive model.

The SAP describes the HI endpoint using six reporting categories. The implemented Mann–Whitney U comparison retained the recorded numerical HI values without top-coding; categorical presentation and the supportive ordinal regression used the reporting categories, grouping recorded values above 5 in the highest category. Mann–Whitney U comparisons were also used for stasis duration and exploratory continuous outcomes. The proportion exceeding 60 seconds of stasis is compared using Pearson's chi-square test. The complication composite uses Pearson's chi-square test without continuity correction. The four confirmatory secondary endpoints use a Bonferroni threshold of 0.0125; exploratory analyses use 0.05 and are hypothesis generating.

Sensitivity analyses assess alternative assignments of missing primary outcomes. Analyses added beyond the SAP, including proportional odds ordinal regression, the 45-second threshold, and additional associations or subgroup explorations, retain their supportive or exploratory labels. They are not retrospectively designated as part of the initial plan. Statistical procedures and interval methods are detailed in the accompanying SAP.

15. Reporting and limitations of available measurements

The revised report follows CONSORT 2025 and separates randomized, complete-case, and specimen-specific denominators. Hemolysis-related repeat venipuncture could not be evaluated because subsequent confirmation orders were not captured in the study's LIS tracking. This outcome is described as unmeasurable, not as having no events.

Adverse-event conclusions are limited to the observation period. Laboratory-defined hemolysis is a preanalytical endpoint; the trial does not establish effects on diagnostic delay, length of stay, or cost. The manuscript and supplementary material distinguish prespecified comparisons from additional analyses performed during revision.

16. Finalization of the plan before enrollment

The initial ethics application was an early description of the proposed study. During preparation for trial registration and bedside implementation, the investigators reviewed the laboratory workflow and specified the final procedures. The investigators confirm that all refinements described below were completed before the first participant was enrolled on 1 June 2026. The dates and contents of the original source documents are preserved; this explanation was compiled for peer review on 8 October 2026.

Randomization and concealment. The initial application proposed triage-stratified randomization with variable blocks of four and six and sealed envelopes. The final plan used an unstratified sequence with fixed blocks of four and allocation through the electronic application, as described in Section 6. Research Randomizer was named in both descriptions.

Specimen for HI assessment. The application initially proposed HI measurements in citrate, SST, and EDTA tubes. Before enrollment, the team learned from the laboratory that HI was available only for SST specimens. The endpoint was consequently restricted to SST; the binary hemolysis threshold was retained.

Timing endpoint. The initial form defined total collection time from cuff inflation to completion of the last tube. The final operational endpoint was cuff inflation to complete cuff release and is reported as venous stasis duration. This distinction is material in the early-release group and is stated explicitly in Section 11.

Secondary outcomes and multiplicity. The application described tube-specific hemolysis, A-DIVA-related comparisons, and ordinal HI, with a three-comparison Bonferroni adjustment. The final SAP defines four secondary endpoints and the corresponding 0.0125 threshold. A-DIVA and tube count are addressed as subgroup variables in the final plan.

Eligibility and collection site. The initial application excluded pregnancy from 28 weeks onwards and focused on antecubital collection. The final eligibility criteria excluded pregnancy at any gestational age and permitted other suitable peripheral collection sites as described in Sections 4 and 8. The final manuscript also explicitly requires at least two ordered tubes.

Cuff-release wording and participant information. The initial application described complete deflation when blood first entered the citrate tube and late release after the third tube. The implemented procedure describes deflation starting at first-tube blood entry and finishing as that tube fills, with late release after the last tube actually collected. The initial methods anticipated that participants would not be informed of the procedural difference, although the consent form described both timings. The final manuscript appropriately reports participants as unblinded.

The final plan is described in the dated SAP and revised manuscript. The investigators report no changes to recruitment or intervention procedures after enrollment began. The additional analyses identified in the revision are distinguished from these pre-enrollment refinements. The source documents and this consolidated description are supplied together so that the reviewer can compare the initial proposal, the final analysis specifications, and the methods actually reported.
