# Supplementary material for: Effect of Early Tourniquet Release on Hemolysis in Emergency Department Phlebotomy: The TOURNI-ED Randomized Trial
Source: Medicina (Kaunas). 2026 Oct 17;62(10):2009. doi: 10.3390/medicina62102009 (PMC13707226; doi:10.3390/medicina62102009)
Supplement: Supplementary file 1 [file medicina-62-02009-s001.zip › Supplementary_Materials_TOURNI-ED.pdf]

Supplementary Material — TOURNI-ED Trial

Early Versus Late Tourniquet Release Under Standardized Cuff Pressure and Specimen Hemolysis in the Emergency Department: A Randomized Controlled Trial

This file contains Table S1, Table S2, Text S3, and Table S4. SAP Version 1.1, dated May 30, 2026, current SAP Version 1.2, the consolidated Study Protocol prepared October 8, 2026, are provided separately in the accompanying reviewer package.

Table S1. Adjusted logistic regression models for the primary outcome (hemolysis index ≥1+; n = 790 complete-case participants).

Covariate	Model 1 (prespecified): OR (95% CI)	P	Model 2 (extended): OR (95% CI)	P
Early release (vs. late release)	0.580 (0.409–0.822)	.002	0.587 (0.413–0.834)	.003
Total A-DIVA score (per point)	1.213 (1.034–1.424)	.018	1.276 (1.105–1.473)	<.001
Triage category, green (ESI 4–5) vs. yellow (ESI 3)	0.720 (0.498–1.040)	.080	0.711 (0.491–1.030)	.071
Number of venipuncture attempts	1.161 (0.832–1.619)	.380	—	—
Age (per year)	—	—	1.000 (0.990–1.011)	.984
Male sex (vs. female)	—	—	1.379 (0.972–1.955)	.071
Body mass index (per kg/m²)	—	—	0.975 (0.942–1.009)	.153
Nurse experience (per year)	—	—	1.016 (0.975–1.059)	.453
Number of tubes collected	—	—	0.974 (0.669–1.418)	.891

Note. Odds ratios (OR) with 95% confidence intervals (CI) and Wald P values from binary logistic regression on the 790 participants with an evaluable hemolysis index (395 per group), analyzed according to randomized allocation. Model 1 is the prespecified model adjusting for total A-DIVA score, triage category, and number of venipuncture attempts. Model 2 is the extended model reported in the main text, adjusting for age, sex, body mass index, total A-DIVA score, nurse experience, triage category, and number of tubes collected. The dependent variable is a hemolysis index of 1+ or greater in the serum separator gel tube. Model 2 includes covariates (age, sex, body mass index, nurse experience, number of tubes collected) that were not listed in the SAP; it is therefore a sensitivity analysis. A-DIVA = modified Adult Difficult Intravenous Access scale; ESI = Emergency Severity Index.

Table S2. Missing-data sensitivity analyses for the primary outcome.

Scenario (imputation of the 2 missing outcomes)	Early release, n/N (%)	Late release, n/N (%)	ARR, percentage points (95% CI)	P

Scenario (imputation of the 2 missing outcomes)	Early release, n/N (%)	Late release, n/N (%)	ARR, percentage points (95% CI)	P
Complete case (n = 790)	68/395 (17.2)	103/395 (26.1)	8.86 (3.12 to 14.54)	.003
Both missing outcomes imputed as no hemolysis	68/396 (17.2)	103/396 (26.0)	8.84 (3.11 to 14.50)	.003
Most favorable to early release (early-release missing = no hemolysis; late-release missing = hemolysis)	68/396 (17.2)	104/396 (26.3)	9.09 (3.35 to 14.77)	.003
Least favorable to early release, the tipping-point scenario (early-release missing = hemolysis; late-release missing = no hemolysis)	69/396 (17.4)	103/396 (26.0)	8.59 (2.84 to 14.27)	.004
Both missing outcomes imputed as hemolysis	69/396 (17.4)	104/396 (26.3)	8.84 (3.09 to 14.53)	.003

Note. Two participants had no evaluable primary outcome (Patient 569, early release; Patient 394, late release; see Table S4). All four possible assignments of the two missing outcomes were examined. The absolute risk reduction (ARR) is the late-release rate minus the early-release rate; confidence intervals were calculated with the Newcombe square-and-add Wilson method and P values with the Pearson chi-square test with Yates continuity correction, as in the primary analysis. The conclusion was unchanged across all scenarios (ARR range, 8.59–9.09 percentage points).

Text S3. Descriptive allocation checks and exploratory analyses.

Allocation checks

The implemented allocation sequence was checked descriptively against the enrollment-ordered assignment record. All 198 permuted blocks contained exactly 4 assignments, and every block contained exactly 2 allocations to early release and 2 to late release (198/198). The recorded assignment matched the analysis data set for all 792 participants (0 mismatches), yielding the planned final balance of 396 participants per group. The maximum cumulative imbalance between groups at any point in the enrollment sequence was 2 participants, reached at enrollment position 14. The block size was not disclosed to clinical, nursing, or research personnel, and the electronic data collection application released each allocation only after eligibility had been confirmed.

Prespecified subgroup analyses

Subgroup analyses of the primary outcome by venous access difficulty (A-DIVA score ≥2 vs. <2) and by number of tubes collected are summarized in Figure 2. Risk ratios (RR) with Katz 95% CIs, computed on the complete-case population:

Subgroup	Early release, n/N (%)	Late release, n/N (%)	RR (95% CI)
Overall	68/395 (17.2)	103/395 (26.1)	0.660 (0.503–0.867)
A-DIVA score ≥2	18/83 (21.7)	32/89 (36.0)	0.603 (0.368–0.988)
A-DIVA score <2	50/312 (16.0)	71/306 (23.2)	0.691 (0.499–0.957)
3 tubes	21/101 (20.8)	27/113 (23.9)	0.870 (0.526–1.439)
4 tubes	46/291 (15.8)	76/280 (27.1)	0.582 (0.420–0.808)

No statistically significant interaction was detected between allocation and venous access difficulty (interaction P = .54) or between allocation and tube count modeled as a continuous variable (interaction P = .31), from Wald tests of the interaction terms in logistic regression models. The 5-tube stratum (5 participants in the primary analysis population) was too small to estimate an effect and is not shown. Hemolysis was more frequent with an A-DIVA score of 2 or greater in both arms.

Exploratory analyses (supportive, not confirmatory)

Within-group association between stasis duration and hemolysis. In the early-release group, median stasis was 37 s (IQR 34–41) in hemolyzed versus 35 s (IQR 32–39) in nonhemolyzed specimens (Mann-Whitney U, P = .084); in the late-release group, 57 s (IQR 52–63) versus 56.5 s (IQR 51–61; P = .182). Point-biserial correlations were 0.089 and 0.059, respectively.

Relation between stasis duration and tube count. Stasis duration was uncorrelated with tube count in the early-release group (Spearman ρ = −0.03; P = .58; median 36, 35, and 42 s for 3, 4, and 5 tubes) but increased strongly with tube count in the late-release group (ρ = 0.64; P < .001; median 48, 59, and 73 s), consistent with the intervention definition.

Forty-five-second stasis threshold. Among the 790 analyzed participants, early-release participants exceeding 45 s of stasis (n = 24), hemolysis occurred in 29.2% versus 16.4% at or below 45 s; among late-release participants (n = 361 exceeding 45 s), rates were 26.3% versus 23.5%. Because threshold crossings are almost entirely separated by group, this comparison is descriptive and confounded with allocation.

Alternative collapsings of the ordinal hemolysis scale. For a threshold of 2+ or greater, hemolysis occurred in 20/395 (5.1%) with early release versus 38/395 (9.6%) with late release (Yates-corrected chi-square, P = .020); for 3+ or greater, 9/395 (2.3%) versus 14/395 (3.5%; P = .397). These analyses were not prespecified and are hypothesis generating.

Table S4. Patient-level account of the 12 laboratory-rejected specimens and their reconciliation with primary-outcome evaluability.

Participant ID	Assigned Arm	Rejected Tube Type	Reason for Laboratory Rejection	SST Tube (Primary Outcome) Status	Primary Analysis Inclusion
Patient 150	Late release (Group B)	K2-EDTA (purple cap)	Clotted specimen	Valid and evaluated (HI = 0)	Included (complete-case)

Participant ID	Assigned Arm	Rejected Tube Type	Reason for Laboratory Rejection	SST Tube (Primary Outcome) Status	Primary Analysis Inclusion
Patient 162	Late release (Group B)	Sodium citrate (blue cap)	Underfilled; incorrect blood-to-citrate ratio	Valid and evaluated (HI = 0)	Included (complete-case)
Patient 265	Late release (Group B)	Sodium citrate (blue cap)	Underfilled; incorrect blood-to-citrate ratio	Valid and evaluated (HI = 0)	Included (complete-case)
Patient 394	Late release (Group B)	SST gel (yellow cap)	Barcode/identity mismatch	Invalid / unmeasurable	Missing (excluded from complete-case)
Patient 510	Early release (Group A)	Sodium citrate (blue cap)	Underfilled; incorrect blood-to-citrate ratio	Valid and evaluated (HI = 0)	Included (complete-case)
Patient 569	Early release (Group A)	SST gel (yellow cap)	Insufficient serum volume	Invalid / unmeasurable	Missing (excluded from complete-case)
Patient 626	Early release (Group A)	K2-EDTA (purple cap)	Clotted specimen	Valid and evaluated (HI = 0)	Included (complete-case)
Patient 635	Early release (Group A)	Sodium citrate & K2-EDTA	Citrate underfilled (incorrect blood-to-citrate ratio); EDTA insufficient volume	Valid and evaluated (HI = 0)	Included (complete-case)
Patient 644	Late release (Group B)	Sodium citrate (blue cap)	Clotted specimen	Valid and evaluated (HI = 0)	Included (complete-case)
Patient 660	Late release (Group B)	K2-EDTA (purple cap)	Clotted specimen	Valid and evaluated (HI = 0)	Included (complete-case)
Patient 661	Late release (Group B)	Sodium citrate (blue cap)	Clotted specimen	Valid and evaluated (HI = 0)	Included (complete-case)
Patient 745	Early release (Group A)	K2-EDTA (purple cap)	Clotted specimen	Valid and evaluated (HI = 0)	Included (complete-case)

Note. Early release (Group A): cuff deflation initiated when blood entered the first tube and completed as that tube filled; late release (Group B): cuff pressure maintained until the last tube filled. SST = serum separator gel tube; HI = hemolysis index.

For 2 patients the index serum separator tube was invalidated by the laboratory (H.394, barcode mismatch; H.569, insufficient serum). Biochemistry results reported from a concurrently collected second separator tube were retained in the dataset for completeness but excluded from the serum biochemistry analyses, which used the same 790-participant population as the primary hemolysis analysis. Venous stasis and complication analyses included all 792 randomized participants.
